# Pharmaceutical Reactivation of Attenuated Apoptotic Pathways Leads to Elimination of Osimertinib Drug-Tolerant Cells

**DOI:** 10.1158/2767-9764.CRC-22-0066

**Published:** 2022-10-31

**Authors:** Matthew J. Martin, Nicolas Floc'h, Matthias Pfeifer, Steven Criscione, Oona Delpuech, Sladjana Gagrica, Yi Yao, Ultan McDermott, Paul D. Smith

**Affiliations:** 1 AstraZeneca Oncology R&D, Cambridge, United Kingdom.; 2 AstraZeneca Oncology R&D, Waltham, Boston, Massachusetts.

## Abstract

Osimertinib is an EGFR tyrosine kinase inhibitor (TKI) with proven clinical efficacy; however, acquired resistance presents an obstacle to curing *EGFR*-driven disease. Recent studies have shown that drug-tolerant persister cells (DTP) have a distinct transcriptional profile that may confer specific vulnerabilities. By definition these cells avoid apoptosis, yet little is known about how their survival is regulated. We found that paradoxically, the proapoptotic gene BIM was upregulated in osimertinib DTPs, and cotreatment with BH3 mimetics could trigger DTP cell death. Furthermore, cIAP proteins, antiapoptotic members of the extrinsic pathway, were significantly elevated in DTPs. cIAP antagonists could block DTP formation as an up-front combination, and could eliminate preformed DTPs. Critically, when treated at the time of maximal osimertinib response, cIAP or MCL1 inhibitor treatment could significantly attenuate the regrowth of *EGFR*m cell line mouse xenografts. Finally, we show that apoptosis can be maximized in cell lines with acquired osimertinib resistance by combining BH3 or SMAC mimetics with agents that target the resistance driver in these models. Taken together, these data suggest novel therapeutic strategies at the point of minimal residual disease or full osimertinib resistance for patients in this critical area of unmet need.

**Significance:**

These studies uncover strategies to use targeted agents that activate apoptosis in non–small cell lung cancer cells that survive initial EGFR TKI treatment.

## Introduction

Precision medicine, whereby specific therapeutic agents are tailored to the particular genetic driver in a given tumor, has revolutionized cancer treatment in the 21st century. One such success story is the discovery of osimertinib, a third-generation EGFR TKI that targets activating mutations with or without the T790M gatekeeper mutation, which has significant clinical benefit in patients with non–small cell lung cancer (NSCLC) with activating mutations in *EGFR* (1). However, like all targeted agents in the metastatic setting, osimertinib treatment rarely leads to outright cures, and invariably resistance develops. Recently, the study of minimal residual disease, a stable period of tumor dormancy after an initial drug response, has intensified. While these tumor cells have not yet developed the genetic alterations that allow unlimited proliferation in the presence of drug, these surviving cells are thought to act as a reservoir that will eventually develop into a resistant tumor. Several research groups have sought to model minimal residual disease *in vitro* by establishing so-called “drug-tolerant persister” (DTP) cells via prolonged treatment of parental cultures with targeted agents (2–4). Previous studies have shown that DTPs undergo profound changes in their epigenome, which drives a distinct program of gene expression to support this unique cellular phenotype. However, little is known about how rare individual cells are able to escape apoptotic death seen in the bulk population after drug treatment. Several studies have pinpointed the induction of BIM, as a consequence of attenuated Ras/MAPK signaling, as the critical step in apoptosis induced by EGFR TKI treatment (5–7). Critically, cell lines resistant to EGFR TKI therapy fail to induce BIM expression, despite suppression of EGFR phosphorylation (8). Nevertheless, the role of BIM in drug tolerance, as opposed to resistance, has yet to be explored.

Apoptosis can be triggered by a myriad of factors, and cells have evolved a complex network of proteins that act to promote or inhibit its execution in response to these stimuli. The cascades regulating apoptosis are generally divided into “intrinsic” – originating within the cell - and “extrinsic” – arising due to binding of extracellular factors to cell surface receptors, which both converge on the activation of cysteine-dependent aspartyl proteases (caspases). Extrinsic apoptosis is primarily triggered by activation of cell death receptors of the TNFα superfamily after binding by their cognate ligands, which generates a protein complex at the cell membrane known as the death-inducing signaling complex (DISC), which can in turn activate caspase-8 (9). To prevent inappropriate activation of this pathway, the inhibitor of apoptosis (IAP) family of proteins can directly bind caspase family members to prevent their activation by sequestration or targeting them for proteasomal degradation (10). A further level of regulation is achieved by the mitochondrial protein SMAC/DIABLO that itself binds and inhibits the IAP family of proteins to promote apoptosis. The discovery of SMAC-mimetic small molecules has allowed the extrinsic pathway to be activated therapeutically, and such compounds have been tested in the clinic as anticancer drugs (11).

In this study, we wished to understand the dynamic status of apoptotic signaling in osimertinib DTPs, with the aim of identifying specific vulnerabilities that could push these cells into cell death. We found that despite initial resistance to apoptosis in DTPs, this phenotype was reversible after prolonged drug removal. We further examined the expression of genes that regulate apoptosis, with a particular focus on BIM, and analyzed the consequences of BIM deletion, and conversely, treatment with BH3 mimetics, on the DTP phenotype. Further analysis showed an upregulation of cIAPs, acting to inhibit the extrinsic apoptosis pathway, and thus we tested the effects of inhibiting cIAP activity via SMAC-mimetic treatment on the establishment and outgrowth of DTPs. Finally, we tested the sensitivity of cell lines with acquired resistance to osimertinib to drugs targeting either the intrinsic or extrinsic apoptotic pathways, with the aim of identifying potential treatment strategies that might have the broadest impact on delaying or preventing EGFR TKI resistance.

## Materials and Methods

### Cell Lines

PC-9 (RRID: CVCL_B260) cells were obtained from European Collection of Authenticated Cell Cultures. IL18 (RRID: CVCL_6659) cells were obtained from Riken Cell Bank. HCC2935 (RRID: CVCL_1265), HCC4006 (RRID: CVCL_1269), HCC827 (RRID: CVCL_2063), and NCI-H1975 (RRID: CVCL_1511). All cell lines were maintained and propagated as monolayer cultures at 37°C in a humidified 5% CO_2_ incubator, and used for ≤10 passages. Cell lines were authenticated at AstraZeneca cell banking using DNA fingerprinting short tandem repeat assays (between 2018 and 2021) and confirmed to be free of *Mycoplasma* through regular testing via an in-house PCR test. Osimertinib-resistant lines were derived by prolonged culture in drug, as described previously (12). Gefitinib-resistant HCC827 cells were created by increased doses of gefitinib to select for those with MET amplification. Doses were increased by approximately 30% every passage until resistant to 500 nmol/L of gefitinib, and then doubled until resistant to 10 μmol/L. PC9 [fPIK3CA (H1047R)] cells were generated as previously described (13). For further details, see Supplementary Table S1.

### Reagents and Western Blotting

All small-molecule inhibitors were synthesized according to published methods. All antibodies used are listed in Supplementary Table S2. For Western blotting culture medium was aspirated from cells and cells were washed once in cold PBS. Cells were scraped into 100 μL lysis buffer [25 mmol/L Tris-HCl (pH 6.8), 3 mmol/L EDTA, 5 mmol/L EGTA, 0.27 mol/L sucrose 0.5% Triton X-100, 50 mmol/L NaF, 2 mmol/L Na_3_VO_4_, 10 mmol/L β-glycerophosphate, 5 mmol/L sodium pyrophosphate, and Complete protease inhibitor tablets (Roche)] per 35-mm dish. Protein concentrations were determined by the bicinchoninic acid (BCA) protocol from Pierce and Western blots were performed by running samples of equal protein concentration on SDS-PAGE gels (NuPAGE Novex 4%–12% Bis-Tris Protein Gel, Thermo Scientific), transferring proteins to nitrocellulose membranes, incubating with primary antibodies overnight, followed by addition of horseradish peroxidase (HRP)-conjugated secondary antibodies (Cell Signaling Technology: goat anti-rabbit RRID: RRID:AB_2099233 and goat anti-mouse RRID: AB_330924) and detected with SuperSignal West Dura chemiluminescent substrate (Thermo Scientific). Where indicated Western blots were quantified using ImageJ software (RRID:SCR_003070).

### Viability Dose–Response Curves

Cells were plated in 96-well plates (1,000 cells/well) and allowed to attach overnight. Cells were then treated with a 9-point, half-log dose response regimen (top dose 10 μmol/L, lowest dose 1 nmol/L), and placed in a 37°C incubator. After 96 hours, wells were incubated with an equal volume of CellTiter Glo reagent, prepared according to manufacturer's instructions, and luminescence immediately read on a SpectraMax iD5 Microplate reader (Molecular Devices). Values were normalized to the average value for DMSO control and plotted using GraphPad Prism 8.4.3 (RRID: SCR_002798) software to calculate IC_50_ values.

### Incucyte Growth Assay

Cells were plated in 48-well dishes (20,000 cells/well) and allowed to attach overnight. Wells were then treated in triplicate with the indicated drugs and placed on the Incucyte S3 bioanalyzer. Wells were washed 1x with PBS and replenished with fresh media containing the appropriate treatments 2x per week. After 10–21 days, depending on the experiment, the dosing regimen was changed as indicated in the results section: briefly, upfront combinations and control osimertinib monotherapy were replaced with drug-free media, while established DTPs were treated with test drug monotherapy or their combination with osimertinib. For experiments determining cell number after drug treatment, cells were cotreated with 1 μmol/L Incucyte NucLight Rapid Red Dye, and readings for red fluorescence signal were taken every four hours.

### Apoptosis Assay

Cells were plated in 96-well dishes (4,000 cells/well) and allowed to attach overnight. Wells were then treated with the indicated drugs in triplicate, along with 1 μmol/L Incucyte Caspase-3/7 green dye. Plates were immediately placed on the Incucyte S3 Bioanalyzer, and readings for confluence and green fluorescent signal were taken every 4 hours. Apoptotic signal was calculated by dividing the number of distinct green spots by confluence, and normalized to the average value for DMSO treatment at 72 hours.

### Senescence-Associated β-Galactosidase Assay

PC9 cells were plated in 12-well dishes (100,000 cells/well) and allowed to attach overnight wells were then treated with the indicated drugs in triplicate. After 10 days DTP cells were washed two times with PBS and replaced with drug-free media. After a further 96 hours, cells were fixed and stained for β-Galactosidase activity, following the manufacturer's instructions (Cell Signaling Technology). After staining, cells were imaged (5 images/well) and the proportion of stained cells was counted manually for each image.

### *In Vivo* Xenografts

All animal studies were conducted in accordance with U.K. Home Office legislation, the Animal Scientific Procedures Act 1986, as well as the AstraZeneca Global Bioethics policy. All experimental work was approved under the framework outlined in project license 70/8894, which has gone through the AstraZeneca Ethical Review Process. Studies in the United States were approved and conducted in accordance with the guidelines established by the internal Institutional Animal Care and Use Committee (IACUC) and reported following the ARRIVE (Animal Research: Reporting In Vivo Experiments) guidelines. Randomization of animals onto study was based on initial tumor volumes to ensure equal distribution across groups. A power analysis was performed whereby group sizes were calculated to enable statistically robust detection of tumor growth inhibition (≥8 per group). PC9 xenografts were established by subcutaneous implantation of 5 × 10^6^, cells per animal, in 100 μL of cell suspension including 50% Matrigel, into the dorsal left flank of female SCID mice (RRID:IMSR_ARC:SCID). For the DFCI-306 PDX model, tumor fragments from donor mice inoculated with primary human lung cancer tissues were harvested and inoculated subcutaneously into the flank of female NSG mice (RRID:IMSR_ARC:NSG). All mice were older than 6 weeks at the time of cell implant. Tumor growth was monitored twice weekly by bilateral caliper measurements and tumor volume calculated using elliptical formula (π/6 × width × width × length). Mice were randomized into vehicle or treatment groups with approximate mean start size of 0.2 cm^3^. Randomization for animal studies was based on initial tumor volumes to ensure equal distribution across groups. Mice were dosed daily by oral gavage with vehicle or 25 mg/kg osimertinib and dosed intravenously once a week for the duration of treatment with 2 mg/kg AZD5582 or 60 mg/kg AZD5991.

Tumor growth inhibition (%TGI) from the start of treatment was assessed by comparison of the geometric mean change in tumor volume for the control and treated groups using the formula: %TGI = (1-{Tt/T0 / Ct/C0} / 1-{C0/Ct}) × 100 where Tt = geo mean tumor volume of treated at time t, T0 = geo mean tumor volume of treated at time 0, Ct = geo mean tumor volume of control at time t and C0 = geo mean tumor volume of control at time 0. Statistical significance was evaluated using a two-tailed *t* test.

### CRISPR Cas9 KO Cell Line Generation

PC9 and HCC2935 cells were engineered by lentiviral transduction of pKLV2-EF1a-Cas9Bsd-W (RRID: Addgene_68343) to stably express Cas9. To generate knockout cell lines, guide RNAs sgNTC (5-GACGCTAAACCAACGGTGC-3), sg BCL2L11#1 (5-TTCTGATGCAGCTTCCATG-3), and sgBCL2L11#2 (5-GCAGGTTCAGCCTGCC-3) were cloned into pKLV2-U6gRNA5 (BbsI)-PGKpuro2ABFP-W (RRID: Addgene_67974) and transduced in Cas9-expressing PC9 and HCC2935 cells. Knockout of target genes and effects on downstream targets were confirmed by Western blot 14 days after transduction.

### RNA Sequencing

*EGFR*m cell lines (PC9, NCI-H1975, HCC827 and HCC2935) were treated with 500 nmol/L osimertinib for 21 days to generate DTP cells. Cells were either harvested immediately or washed two times with PBS and then replaced with drug-free media for a further 24 hours (short recovery), or for a prolonged time until exponential cell proliferation was evident (long recovery; PC9: 7 days; NCI-H1975: 3 days; HCC827: 4 days; HCC2935: 10 days) and then harvested. In parallel, parental cell lines were grown for 21 days, then treated with 500 nmol/L or vehicle control for 24 hours and harvested. Cells were lysed in RLT buffer (Qiagen) and RNA extracted using the Qiacube HT according to manufacturer's instructions, and RNA concentration quantified using the Qubit fluorometer. Illumina mRNA TruSeq library was used and sequenced on 9 lanes of Illumina HiSeq4000 with paired-end 150 bp by the CRUK Genomics Core Facility. Analysis of this data was as previously described (14); global analysis of this dataset is reported in a parallel study (Criscione and colleagues, manuscript under review).

### Statistical Analyses

Data were expressed as mean ± SE. Differences were tested by two-tailed *t* tests. The values *P* < 0.05 were considered statistically significant. Statistical analysis was done using GraphPad Prism software (*t* test). For the *in vivo* experiment, data were analyzed when at least 6 of 8 animals remained in the study (Fig. 4) or at the end of study (Fig. 5E).

### Data Availability Statement

Gene expression data generated in this study are taken from a dataset publicly available through the NCBI GEO database under accession code GSE193259.

All other data generated in this study are available within the article and its Supplementary Data files.

## Results

**FIGURE 1:**
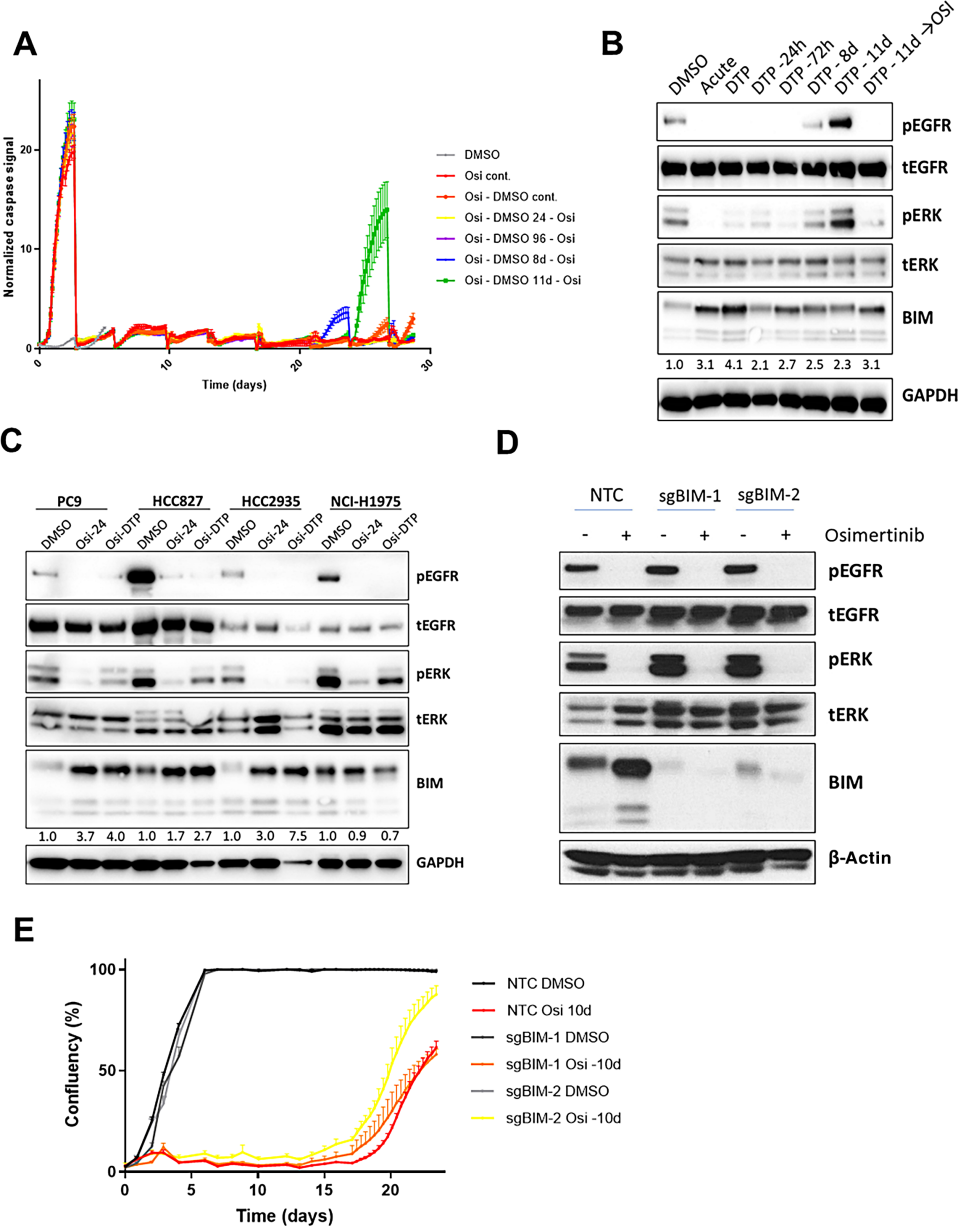
Osimertinib drug tolerant cells re-aquire apoptotic capacity after drug holiday. **A,** Caspase activity in PC9 cells treated with osimertinib (500 nmol/L) for 10 days to generate DTPs, followed by the indicated periods of drug-free media (DMSO) prior to rechallenge with osimertinib. **B,** Western blot showing relative expression of BIM protein in PC9 cells after acute (24 hours) osimertinib (500 nmol/L) treatment compared with DTPs (10-day treatment) or DTPs released from drug for the indicated times. Numbers represent fold-increase in BIM expression by densitometry, compared with DMSO, normalized to GAPDH expression. **C,** Western blot showing relative expression of key protein markers after acute (24 hours) or chronic (DTP; 14 days) osimertinib treatment in a panel of *EGFR*m cell lines. Numbers represent fold-increase in BIM expression by densitometry, compared with DMSO, normalized to GAPDH expression. **D,** Western blot showing expression of BIM protein in PC9-derived cell lines with CRISPR-mediated BIM deletion, compared with non-targeting control (NTC) cells treated with or without osimertinib (160 nmol/L) for 24 hours. **E,** Cell confluence over time in BIM-deleted cells versus control after osimertinib (500 nmol/L) treatment for 10 days, followed by release from drug.

It has been previously shown that the treatment of PC9 cells (*EGFR* ex. 19 del activating mutation) with EGFR TKIs results in the death of a majority of cells, but that a subset of cells survives as DTPs (2). However, there is some debate as to whether the ability of cancer cells to persist treatment with targeted agents is a heritable phenomenon (15), or if their survival response is stochastic in nature and regrown cultures reestablish a heterogeneous drug response (16, 17). Thus, we tested the osimertinib-driven induction of apoptosis over time in PC9 DTPs, while varying the duration of release from drug prior to rechallenge (Fig. 1A), using the Incucyte imaging platform. Interestingly, when allowing 24–96 hours of recovery time, DTPs showed no further induction of caspase-3/7 activation after osimertinib retreatment, while increasing recovery time to 8 days only resulted in a minimal induction of apoptosis by drug. However, extending the recovery time to 11 days, a time point where cells were in an exponential growth phase (Supplementary Fig. S1A), osimertinib could induce robust caspase activation. This observation argues that osimertinib treatment of PC9 cells does not select for a preexisting subclone that is resistant to EGFR-TKI–induced cell death, and demonstrates that once the pressure of drug treatment is taken away, growing cultures reestablish the propensity for apoptosis induction. Subsequent experiments quantifying cell number using a nuclear-staining dye found that up to 9.1% of the original PC9 cell population survived as DTPs after 14 days, implying that the ability to survive drug is a relatively widespread characteristic in the population (Supplementary Fig. S1B). Furthermore, we observed that reestablishment of EGFR phosphorylation and downstream signaling (as measured by ERK phosphorylation), correlated with apoptotic response, as we observed partial recovery of these signaling markers at 8 days, and full restoration by 11 days post-drug release (Fig. 1B).

Variations in gene expression are thought to underpin heterogeneous responses to targeted therapy (17). We wished to understand the relative expression levels of the pro-apoptotic protein BIM, which has previously been implicated in a key mediator of apoptosis downstream of EGFR TKI treatment (5). We hypothesized that DTPs would show lower levels of BIM, reflecting their impaired ability to trigger the apoptotic cascade. However paradoxically, DTPs from a panel of *EGFR*m cell lines (with the exception of NCI-H1975 cells) showed high levels of BIM protein, greater even than acute (24 hours) osimertinib treatment of parental cells (Fig. 1C). Although BIM is regulated primarily at the protein level downstream of EGFR via ERK activity (18), we nevertheless also observed upregulation of the corresponding transcript (*BCL2L11*) in PC9 and HCC2935 DTPs, when examining a DTP RNA-sequencing dataset we generated (Supplementary Fig. S1C). Interestingly, levels of BIM were maintained at an elevated plateau level over the course of the 11-day period of drug release, (Fig. 1B; between 2.1 and 2.7-fold increase over the original untreated population), and were not markedly further induced when these released cells were rechallenged with osimertinib. Conversely, we found that BIM upregulation was not required for the DTP phenotype, as deletion of BIM via CRISPR-mediated gene editing (Fig. 1D) failed to block the establishment of DTPs, in fact resulting in an increased number of surviving cells after osimertinib treatment (Fig. 1E; Supplementary Fig. S1D). BIM knockout PC9 cells showed a blunted induction of active caspase 3/7 compared with control cells, which likely underpins the observed increase in DTP numbers (Supplementary Fig. S1E).

**FIGURE 2:**
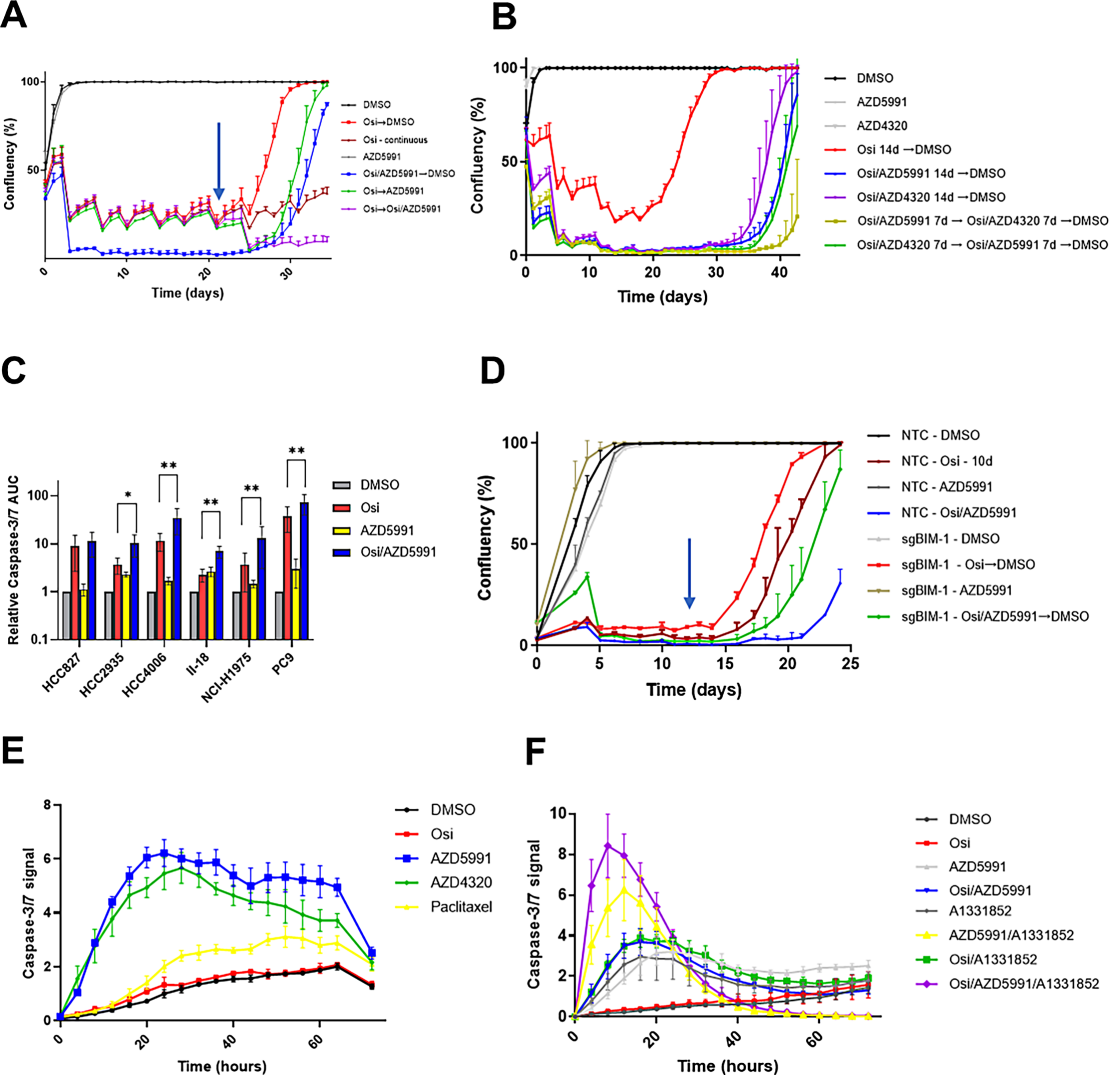
BH3 mimetics can trigger apoptosis in established DTPs. **A,** PC9 DTP assay measuring effect of AZD5991 (300 nmol/L) in up-front combination (blue), DTP monotherapy (green) and DTP combination (violet) vs. osimertinib (500 nmol/L) monotherapy controls (red). Arrow indicates change in dosing regimen. **B,** PC9 osimertinib DTP assay measuring the effect of alternating AZD4320 or AZD5991 (300 nmol/L each) + osimertinib combination dosing schedules, as indicated. **C,** Caspase-3/7 activity in a panel of *EGFR*m cells treated for 72 hours with osimertinib (160 nmol/L), AZD5991 (300 nmol/L) or their combination, compared with control. Values represent AUC of caspase-3/7 signal for the duration of the experiment. **D,** DTP assay measuring the effect of the upfront combination of osimertinib (500 nmol/L) with AZD5991 (300 nmol/L) in control (NTC) and BIM-deleted PC9 cells for the indicated duration. Arrow indicates change in dosing regimen. **E** and **F,** Caspase-3/7 activity in established PC9 DTPs treated with the indicated drugs (osimertinib 500 nmol/L; AZD5991 300 nmol/L; AZD4320 300 nmol/L; paclitaxel 30 nmol/L; A1331852 100 nmol/L; K-975 100 nmol/L).

The family of prosurvival BCL2 family proteins, which includes MCL1, BCL2 and Bcl-xL, act to inhibit apoptosis by binding to BIM, preventing it from interacting with the pro-apoptotic proteins Bax and Bak to initiate cytochrome C release and trigger the apoptotic cascade (19). We hypothesized that these BCL2 family proteins could be blocking this process in DTPs, and thus these cells would be sensitive to inhibitors such as AZD5991 (MCL1; ref. 20) or AZD4320 (BCL2/Bcl-xL; ref. 21). First, we performed the DTP assay in PC9 cells with three distinct dosing regimens to understand the effects of AZD5991 and AZD4320 on DTP biology (Fig. 2A; Supplementary Fig. S2A and S2B). In the first strategy, parental cells were treated with the upfront combination of osimertinib and the BH3 mimetic (osimertinib + AZD5991 or osimertinib + AZD4320) to assess how this affects the establishment of a DTP population (blue lines). In this instance, both AZD5991 and AZD4320 significantly inhibited the number of established DTPs. However, upon drug removal a population of surviving cells was revealed, which over time could regrow to populate the well, indicating these combinations could not completely eliminate DTPs. We further postulated that BLC2/Bcl-xL activity could compensate for inhibition of MCL1, and vice versa, in terms of suppressing full apoptosis induced by osimertinib. This hypothesis is supported by the observation that a 7-day AZD4320 combination after 7 days of the AZD5991 combination showed a greater ability to prevent the establishment of DTPs than 14 days of the AZD5991 combination (Fig. 2B; Supplementary Fig. S2C), despite combinations with AZD5991 being more potent that those with AZD4320 (Fig. 2B; Supplementary Fig. S2B). The effects of these inhibitors were not limited to PC9 cells, as AZD5991 and to a lesser extent AZD4320 could significantly impair the establishment of DTPs from a panel of *EGFR*m cell lines when given as an upfront combination (Supplementary Fig. S2D and S2E). This is likely due to enhanced apoptosis, as both BH3 mimetics enhanced osimertinib-driven apoptosis with acute treatment in a panel of *EGFR*m cell lines (Fig. 2C; Supplementary Fig. S2F). Interestingly, AZD5991 is able to enhance osimertinib-driven apoptosis in BIM knockout cells (Supplementary Fig. S2G), and accordingly impairs the establishment of DTPs (Fig. 2D; Supplementary Fig. S2H), albeit less effectively than in control cells.

To assess the effects of MCL1 or BCL2/BcL-xL inhibition on established DTPs, PC9 cells were treated with osimertinib monotherapy for 21 days, at which time cells were washed and retreated with either BH3 mimetic monotherapy, or in combination with osimertinib. DTP monotherapy (green lines) with either AZD5991 (Fig. 2A) or AZD4320 (Supplementary Fig. S2B) resulted in an initial period of decreased cell number, followed by an increase in growth, despite maintaining drug treatment indefinitely. When DTPs were treated with osimertinib/BH3 mimetic combinations (violet lines), this resulted in a similar decrease in cell number to monotherapy treatment, at which time cell number reached a plateau. These initial decreases in cell number were likely due to elimination of a proportion of this DTP population by apoptosis, because we observed that AZD5991 and AZD4320 were able to induce significant levels of caspase-3/7 activation at the DTP stage, to a much greater extent than the cytotoxic chemotherapy paclitaxel (Fig. 2E), despite paclitaxel inducing robust caspase activity in parental cells at the same dose (30 nmol/L; Supplementary Fig. S2I). To test the possibility that inhibiting multiple prosurvival BCL2 family proteins is required to eliminate all DTPs, we combined osimertinib, AZD5991, and AZD4320 together in an up-front dosing regimen. However, the combination of AZD5991 and AZD4320 alone proved highly toxic in parental cells, as did a similar combination of AZD5991 with the Bcl-xL–selective compound A-1331852 (Supplementary Fig. S2J and S2K). Nevertheless, established DTPs were highly sensitive to the AZD5991/AZD4320 or AZD5991/A-1331852 combination, with or without cotreatment with osimertinib, indicating DTPs can potentially be eliminated by maximizing the induction of apoptosis (Fig. 2F). We further combined AZD5991 and the BCL2-specific drug venetoclax in the DTP assay; however, we found that the addition of venetoclax to AZD5991 failed to impact the previously observed effects of AZD5991 alone on DTP biology (Supplementary Fig. S2L). It has recently been demonstrated that in osimertinib DTPs there is increased nuclear localization of the YAP transcriptional regulator, where it can complex with TEAD and other transcription factors to attenuate the apoptotic response (22). Accordingly, this study showed that a novel small-molecule inhibitor of TEAD could enhance osimertinib-driven apoptosis in sensitive parental cells. We confirmed these results using K-975, a recently reported covalent inhibitor of TEAD (23), in the osimertinib-naïve setting (Supplementary Fig. S2M). Interestingly, K-975 could induce caspase activation in established DTPs when combined with osimertinib, with similar kinetics to AZD5991 (Supplementary Fig. S2N). Critically, K-975 treatment had only marginal effects on DTP apoptosis when given as a monotherapy, highlighting the requirement for maintaining suppression of EGFR signaling in order for TEAD inhibition to result in cell death. Overall, we conclude that combining osimertinib with BH3 mimetics can significantly impair the establishment and survival of DTPs, but residual cells after EGFR TKI treatment are not completely eliminated by doublet combinations.

**FIGURE 3:**
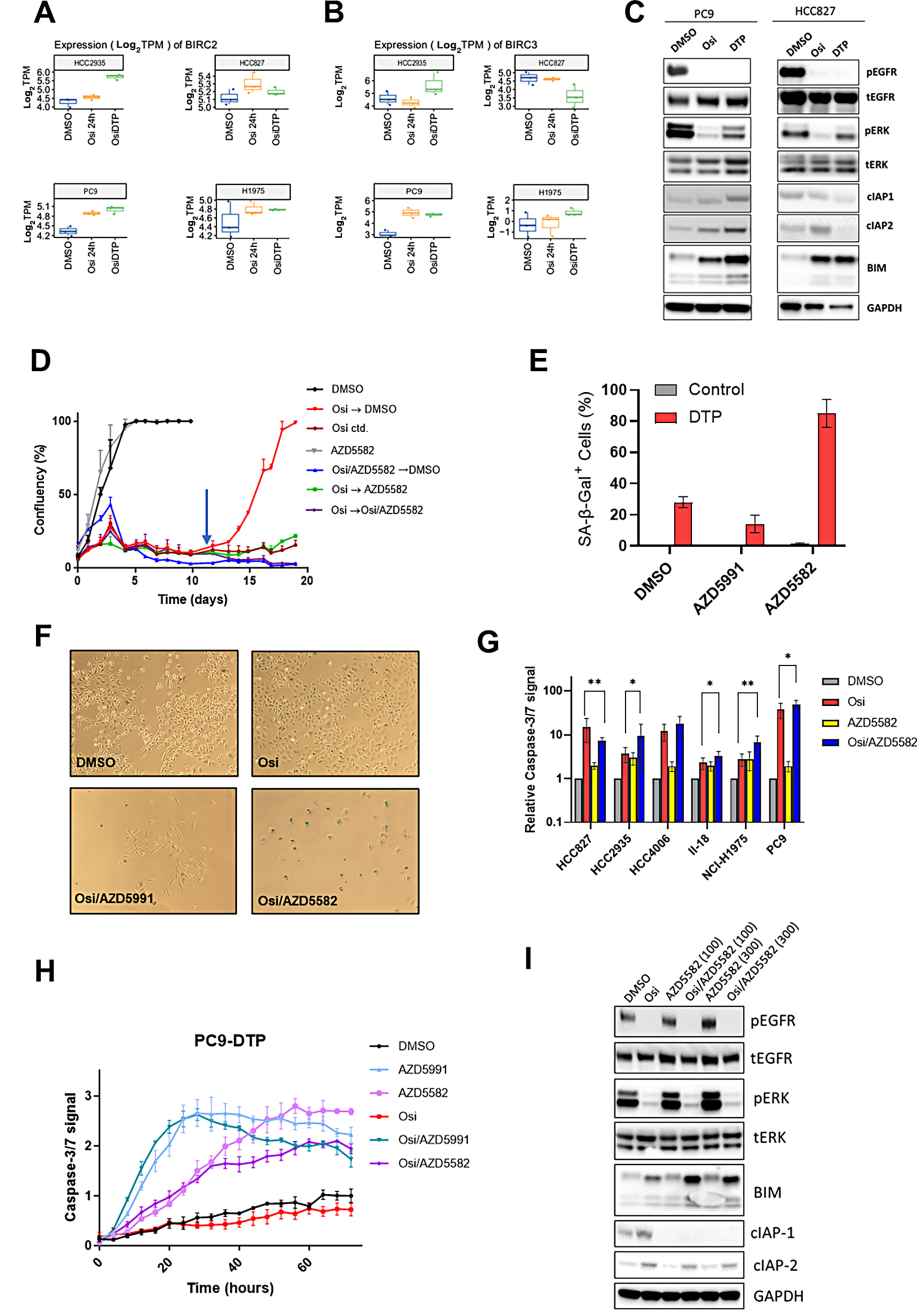
DTPs upregulate cIAP proteins and are sensitive to SMAC-mimetic treatment. **A/B,** mRNA expression of *BIRC2/BIRC3* transcripts taken from RNA-sequencing dataset in panel of *EGFR*m cell lines treated acutely (24 hours) or chronically (DTP; 21 days) with 500 nmol/L osimertinib. **C,** Western blot showing relative expression of the indicated proteins after acute (24 hours) osimertinib treatment compared with DTPs. **D,** PC9 DTP assay measuring effect of AZD5582 (100 nmol/L) in up-front combination (blue), DTP monotherapy (green) and DTP combination (violet) versus osimertinib (500 nmol/L) monotherapy controls (red). Arrow indicates time of change in dosing regimen. **E,** Proportion of cells positively staining for senescence-associated β-galactosidase (SA-β-Gal) after 10-day treatment with the indicated osimertinib combinations (DTP) followed by 96 hours of drug-free media, compared with vehicle and monotherapy controls. **F,** Representative images following SA-β-Gal staining of the indicated treatment groups from the experiment described in **E**. **G,** Caspase activity in a panel of *EGFR*m cells treated for 72 hours with osimertinib (160 nmol/L), AZD5582 (100 nmol/L) or their combination, compared with control. Values represent AUC of caspase signal for the duration of the experiment. **H,** Caspase-3/7 activity in established PC9 DTPs treated with the indicated drugs (osimertinib 500 nmol/L; AZD5991 300 nmol/L; AZD5582 100 nmol/L). **I,** Western blot showing relative expression of the indicated proteins in PC9 cells treated with the drugs (osimertinib 160 nmol/L; AZD5582 100 nmol/L or 300 nmol/L) for 24 hours.

To identify other cell death mechanisms that could be governing the survival of DTPs, we returned to our RNA-sequencing dataset, and found that *BIRC2* and *BIRC3* were elevated in DTPs derived from the PC9, HCC2935 and NCI-H1975 cell lines (Fig. 3A/B). These transcripts correspond to the inhibitor of apoptosis proteins cIAP1 and cIAP2 which are critical components of the extrinsic apoptotic pathway downstream of TNF family receptors. Specifically, these IAPs act as E3 ligases that promote ubiquitination and degradation of caspases. This elevated mRNA translated to increases in cIAP1 and cIAP2 protein in PC9 DTPs (3.9- and 5.7-fold increase, respectively; Fig. 3C). Furthermore, we observed *BIRC2/3* mRNA upregulation in DTPs derived from a majority (3/4) of *EGFR*m cell lines (Fig. 3A and B), and upregulation of cIAP1/2 in HCC2935 at the protein level (Supplementary Fig. S3A). To determine whether PC9 DTPs were vulnerable to inactivation of cIAP function, we performed the DTP assay with the bivalent SMAC-mimetic AZD5582 which acts to prevent cIAP activity and promote caspase activation. Similar to cotreatment with BH3 mimetics, AZD5582 significantly decreased the number of PC9 (Fig. 3D), HCC2935 (Supplementary Fig. S3B), and NCI-H1975 (Supplementary Fig. S3C) DTPs that could establish when given as an up-front combination. However, in contrast to previous observations with AZD5991 or AZD4320, osimertinib/AZD5582-treated DTPs failed to regrow upon drug withdrawal (Fig. 3D; Supplementary Fig. S3D and S3E), indicating this combination is strongly antagonistic to the proliferative potential of these cells. Further analysis showed that any surviving DTPs from this combination had a very high frequency of positive staining for β-galactosidase, compared cells treated with the AZD5991 combination or osimertinib monotherapy, indicating the induction of a senescent phenotype (Fig. 3E and F). Moreover, AZD5582 monotherapy treatment of established DTPs (green line; Fig. 3D) maintained the DTPs at low levels that did not increase over time. These effects of AZD5582 were corroborated using an alternative, monovalent SMAC-mimetic LCL161 (Supplementary Fig. S3F). Interestingly, HCC827 cells failed to upregulate *BIRC2/3* mRNA (Fig. 3A/B) or cIAP1/2 protein (Fig. 3C), and were largely unaffected by cotreatment with AZD5582 (Supplementary Fig. S3G). As expected, treatment with AZD5582 enhanced the induction of apoptosis by osimertinib in multiple *EGFR*m cell lines (Fig. 3G). Furthermore, all SMAC mimetics tested could enhance osimertinib-induced caspase-3/7 activation in PC9 cells (Supplementary Fig. S3H). Commensurate with its ability to prevent outgrowth of established DTPs, AZD5582 was able to specifically induce caspase activation in PC9 DTPs with a similar magnitude to MCL1 inhibition via AZD5991 treatment, albeit with delayed kinetics (Fig. 3H). Interestingly, while AZD5582 is reported to bind and promote degradation of both cIAP1 and cIAP2 (24), we found that AZD5582 could only block osimertinib-induced upregulation of the former, but not the latter after 24-hour treatment (Fig. 3I). We further observed that osimertinib/AZD5582 combination treatment caused a greater degree of BIM upregulation compared with osimertinib monotherapy (Fig. 3G), implying a potential cooperation between the intrinsic and extrinsic apoptotic pathways, and indeed we found that combining AZD5991 and AZD5582 on established DTPs led to potent inhibition of regrowth (Supplementary Fig. S3I).

**FIGURE 4:**
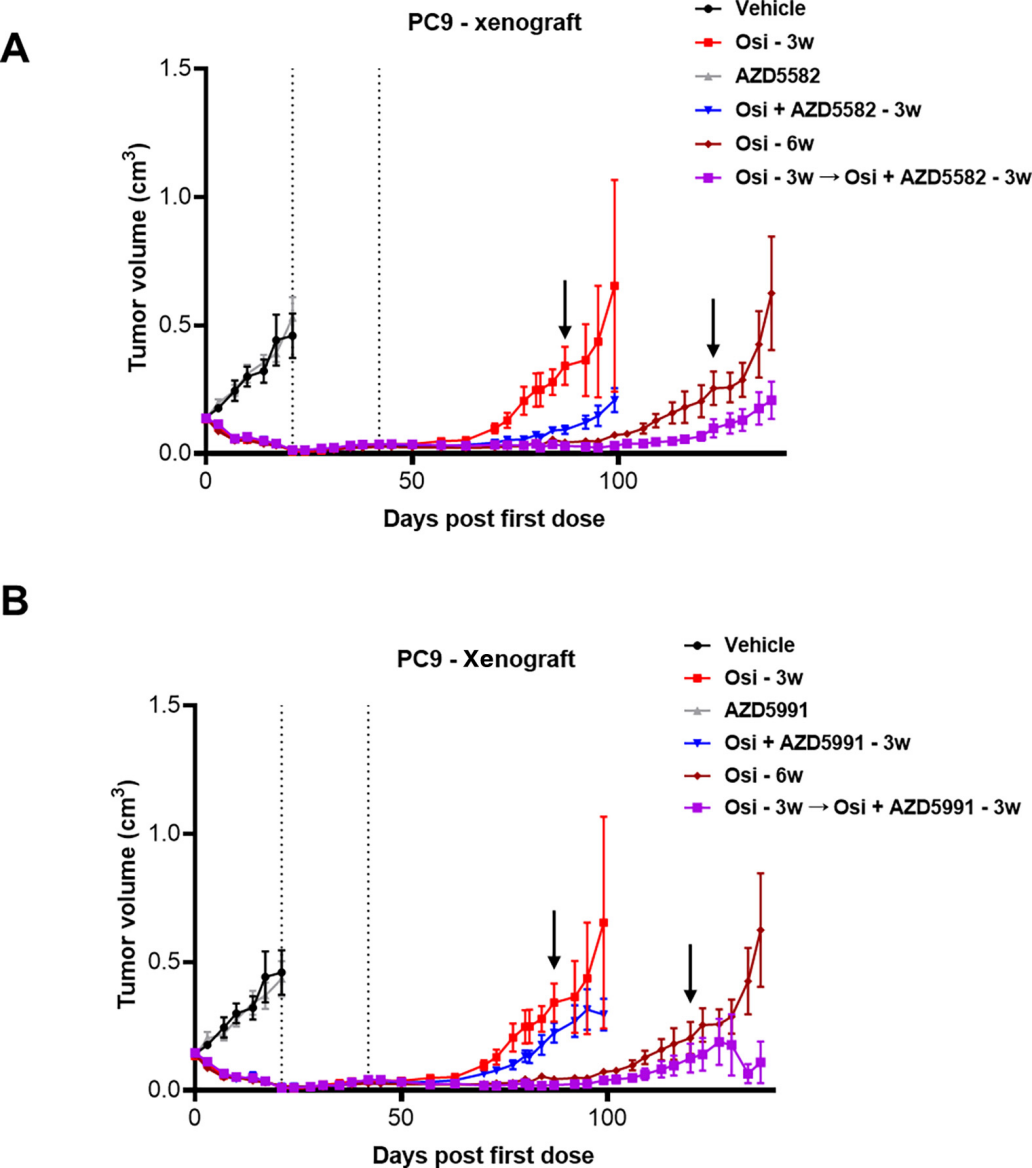
SMAC mimetics inhibit DTP growth *in vivo*. **A,** Growth curves of PC9 xenograft tumors treated with osimertinib (25 mg/kg), AZD5582 (2 mg/kg) or their combination for 21 days, followed by a period of regrowth. Concurrently, tumors were treated for 21 days with osimertinib followed by a further 21 days of osimertinib alone or in combination with AZD5582 prior to the regrowth phase. **B,** Xenograft growth curves as in **A**, plotting the data for AZD5991 (60 mg/kg) treatment instead of AZD5582.

To model the effects of BH3- or SMAC-mimetic treatment on the persister phenotype *in vivo*, we employed a PC9 xenograft model, and pursued two distinct treatment strategies. The first tested the effects of the combination of osimertinib + AZD5991 or AZD5582 combination, in comparison to osimertinib monotherapy. Because osimertinib monotherapy has such strong efficacy in this model, we employed a dosing regimen that included a regrowth phase after 3 weeks of therapy, from which we could infer the relative extent of residual disease at the time of maximal response. To model effects on DTPs themselves, we first treated with osimertinib for 3 weeks, followed by treatment with either osimertinib + cell death drugs or continued osimertinib monotherapy for a further 3 weeks, and finally a regrowth phase. We observed that the up-front combination of osimertinib + AZD5582 achieved a modest but significant delay in tumor regrowth (Fig. 4A; Supplementary Fig. S4A, *P* < 0.01, two-tailed *t* test), suggesting a reduction in DTP number in the *in vivo* setting. However contrary to the *in vitro* data, the AZD5991 combination resulted in no significant delay in regrowth (Fig. 4B; Supplementary Fig. S4A). Following the second strategy of testing the AZD5582 combination at the DTP phase again resulted in a modest but significant delay in tumor regrowth (Fig. 4A; Supplementary Fig. S4B; *P* < 0.05, two-tailed *t* test). MCL1 inhibition via AZD5991 cotreatment at the DTP stage revealed a trend toward delayed regrowth, although this failed to reach statistical significance (Fig. 4B; Supplementary Fig. S4B; note that apparent decrease in tumor size at the end of study in this group is due to removing two mice with large tumors due to poor condition; Supplementary Fig. S4C). Taken together, these data show that combining osimertinib with SMAC-mimetic treatment can reduce the number of DTPs that can initially establish, and further inhibited established DTPs after a period of osimertinib monotherapy, albeit at a lower magnitude than is seen *in vitro*.

**FIGURE 5:**
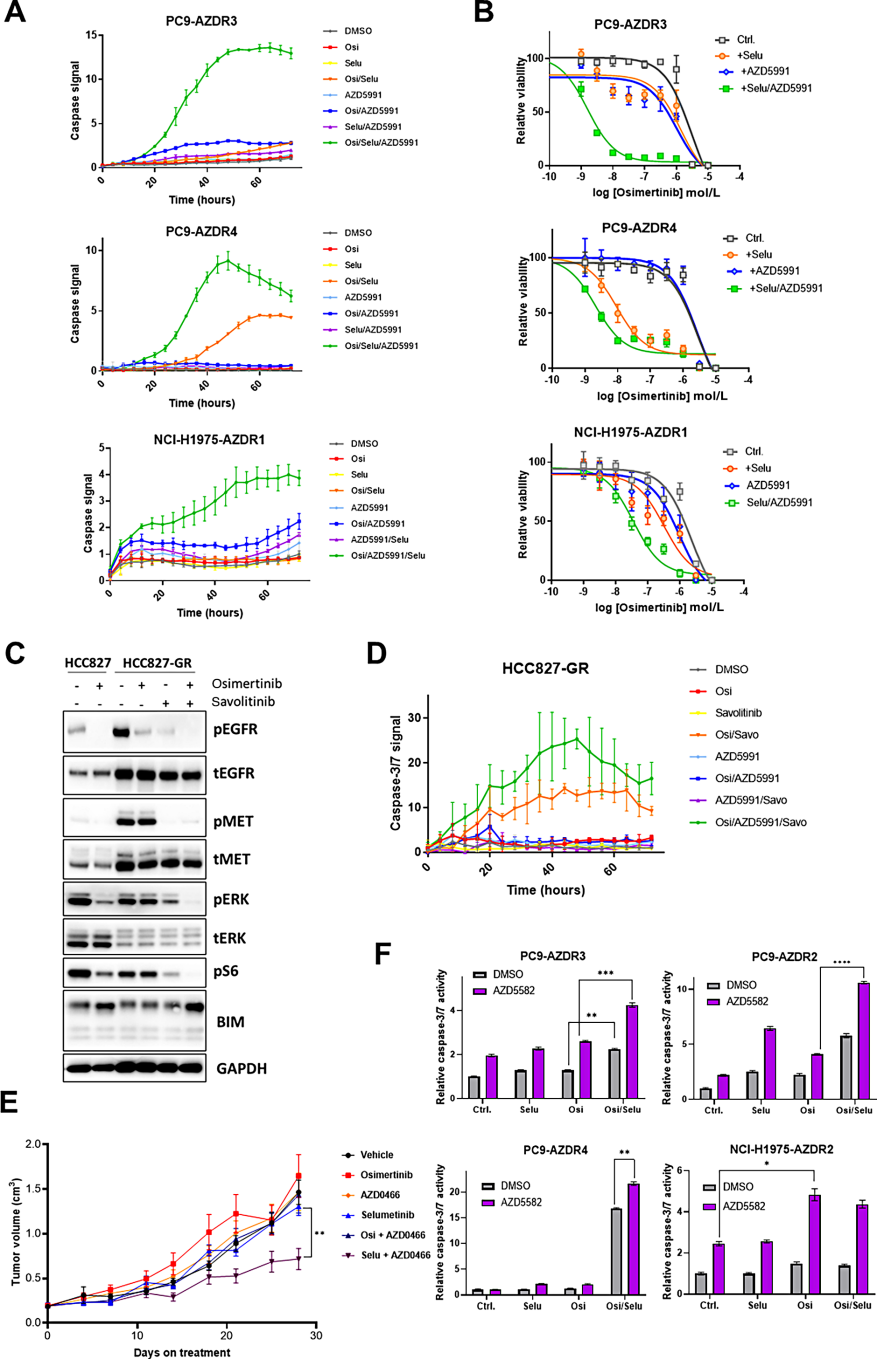
BH3 and SMAC mimetics enhance apoptosis induced by agents targeting resistance drivers. **A,** Caspase-3/7 activity in PC9-AZDR3, PC9-AZDR4, and NCI-H1975-AZDR1 cells treated for 72 hours with osimertinib (160 nmol/L), selumetinib (300 nmol/L), and AZD5991 (300 nmol/L), alone or in combination as indicated, compared with control. **B,** Osimertinib dose–response curves in PC9-AZDR3 PC9-AZDR4 and NCI-H1975-AZDR1 cells, cotreated with the indicated compounds at a single dose (300 nmol/L). **C,** Western blot analysis showing relative expression of the indicated proteins in HCC827 and HCC827-GR cells treated with osimertinib (160 nmol/L), savolitinib (1 μmol/L) or their combination as indicated for 24 hours. **D,** Caspase-3/7 activity in HCC8727-GR cells treated for 72 hours with the indicated compounds (osimertinib 160 nmol/L; savolitinib 1 μmol/L; AZD5991 300 nmol/L; AZD8835 1 μmol/L), compared with control. **E,** Growth curves of DFCI-306 PDX tumors treated with the indicated drugs [osimertinib 25 mg/kg; AZD0466 30 mg/kg once weekly (QW); selumetinib 12.5 mg/kg twice daily (BID) 4 days on/3 days off] for 28 days. **F,** Caspase activity in a panel of *EGFR*m cells treated for 72 hours as indicated [osimertinib 160 nmol/L; selumetinib 300 nmol/L; AZD5582 (100 nmol/L)], compared with control. Values represent AUC of caspase signal for the duration of the experiment. Two-tailed *t*-tests were used for statistical analyses. ****, *P* < 0.0001; ***, *P* < 0.001; **, *P* < 0.01; *, *P* < 0.05.

Drug resistance, as opposed to tolerance, is defined by the ability of cells to avidly proliferate in the presence of drug, after acquisition of heritable changes to their genome or epigenome. To model the progression of tumors that have relapsed on osimertinib treatment, we generated a panel of resistant cell lines derived by prolonged culture of parental *EGFR*m lines in drug, until proliferation was established. Previous studies showed that osimertinib-resistant clones often develop a dependency on the MAPK pathway, and are sensitive to pharmacologic inhibition of MEK (12, 25). For this study, we wished to determine whether these acquired resistant lines showed enhanced sensitivity to BH3 mimetics in the presence or absence of osimertinib cotreatment. However, while we observed a small shift towards increased sensitivity to MCL1 inhibition via AZD5991 in some resistant clones (Supplementary Fig. S5A), this was not seen in all clones, nor were there increased sensitivities to the BCL2/Bcl-xL inhibitor AZD4320 (Supplementary Fig. S5B). However, we hypothesized that the BH3-mimetic drugs might cooperate with the MEK inhibitor selumetinib in resistant clones that had been shown to have sensitivity to inhibition of the MAPK pathway, to maximize apoptosis. To investigate this, we employed the previously established (12) osimertinib-resistant clones PC9-AZDR3, PC9-AZDR4, and NCI-H1975-AZDR1 that have been shown to cells treated with osimertinib, selumetinib, or a BH3-mimetic drug, either alone or together. Both PC9-AZDR4 and NCI-H1975-AZDR1 have been shown to have enhanced sensitivity to selumetinib, though that study could not identify a genetic mechanism driving resistance in these lines. Importantly, we observed significantly increased caspase-3/7 activation in cells treated with the osimertinib, selumetinib and AZD5991 triplet compared with any possible doublet treatment (Fig. 5A). As previously observed (12), PC9-AZDR4 cells could be sensitized to osimertinib through selumetinib cotreatment (Fig. 5B); however, MCL1 inhibition via AZD5991 cotreatment did not alter the response to EGFR TKI treatment. Critically, adding AZD5991 to selumetinib further enhanced the osimertinib sensitization effect (reducing IC_50_ from ∼9 nmol/L to ∼2 nmol/L). Moreover, despite not showing a sensitization effect with selumetinib alone, PC9-AZDR3 cells could be dramatically sensitized to osimertinib with selumetinib + AZD5991 cotreatment (Fig. 5B). Adding AZD4320 to the osimertinib/selumetinib combination could also significantly enhance caspase-3/7 activation, albeit to a more modest degree than MCL1 inhibition (compare Supplementary Fig. S5C with Fig. 5A).

We further hypothesized that alternative resistance mechanisms could be targeted in such a manner. To model this, we employed an HCC827 clone that had acquired resistance to erlotinib via MET amplification (Fig. 5C). Interestingly, these cells do not show induction of BIM upon treatment with monotherapy of either osimertinib or the MET inhibitor savolitinib; however, combining these two agents leads to enhanced BIM expression. We tested their response to osimertinib when co-dosed with savolitinib, with or without MCL1 inhibition via AZD5991 cotreatment. Again, we observed an analogous effect to the selumetinib combinations described above, whereby caspase-3/7 activation was significantly enhanced by the AZD5991 triplet, above the levels seen with the savolitinib/osimertinib doublet (Fig. 5D). Finally, we employed a cell line in which we have established osimertinib resistance, by knocking in the PIK3CA (H1047R) activating mutation (13), that is partially sensitive to the PIK3CA inhibitor AZD8835. Critically, we see that while AZD8835 enhances osimertinib-induced apoptosis in these cells, the AZD5991-based triplet combination significantly accelerates the onset and increases the magnitude of caspase activation (Fig. 5D), data which reflects the ability of the triplet to effectively prevent cell growth in a 10-day assay (Supplementary Fig. S5D). Furthermore, commensurate with its ability to enhance apoptosis in osimertinib-sensitive parental cells (Supplementary Fig. S2M), we find that TEAD inhibition via K975 cotreatment can significantly inhibit proliferation (Supplementary Fig. S5E) and enhance cell death (Supplementary Fig. S5F) when combined with inhibitors of resistance pathways, such as selumetinib in NRAS-mutant cells, AZD8835 in PIK3CA (H1047R)-expressing cells or savolitinib in MET-amplified cells – the latter in agreement with previous studies (22). Taken together these data indicate that by maximizing the apoptotic effect in resistant cells, we can potentially eliminate residual survival through the addition of apoptosis potentiating inhibitors.

To model the sensitization effect of MEK inhibition combined with BH3-mimetic treatment *in vivo*, we utilized a PDX model derived from a patient who had relapsed after a period of osimertinib therapy. This PDX model carries an acquired V600E mutation in BRAF, and represents the putative resistance driver. Unfortunately, due to limiting toxicities, we were unable to codose osimertinib, selumetinib, and AZD0466 (a dendromer conjugate of AZD4320), however doublet therapy of these drugs was achievable in this *in vivo* system. As expected, these tumors were completely resistant to osimertinib monotherapy (Fig. 5E). Similarly, neither selumetinib nor AZD0466 monotherapy significantly altered the growth of these tumors. Critically, selumetinib combined with AZD0466 led to significant tumor growth inhibition (45% compared with selumetinib alone and 51% compared with AZD0466 alone at day 28 of treatment). Thus, combining BH3-mimetic therapy with agents targeting the driver resistance mechanism in relapsed patients could represent an effective treatment strategy.

Finally, we aimed to assess the consequences of SMAC-mimetic treatment in the osimertinib-resistance setting. In NCI-H1975-AZDR2 cells, in contrast with BH3 mimetics, AZD5582 monotherapy could induce significant caspase-3/7 activation, which was further enhanced by osimertinib cotreatment (Fig. 5F). In PC9-resistant clones, selumetinib treatment could promote modest caspase activation, which was enhanced by osimertinib cotreatment. Further induction of apoptosis could be achieved by triplet treatment with osimertinib/selumetinib and AZD5582 in resistant clones with sensitivity to MEK inhibition (Fig. 5F), similar to what was observed for MCL1 inhibition (Fig. 5A), which as expected translated to a greater sensitization to osimertinib in proliferation assays (Supplementary Fig. S5G).

## Discussion

Elimination of transformed cells from a growing tumor is the ultimate goal for all cancer therapeutics. By inducing cell death, the reservoir for cells that can eventually acquire genetic or nongenetic alterations allowing proliferation in drug will be reduced. EGFR TKIs have been remarkably successful for treating patients with NSCLC bearing *EGFR* activating mutations, and preclinical evidence in cell lines show these inhibitors potently induce apoptosis. However, these drugs are generally not curative, and resistant tumors arise from a pool of cells that survive the initial therapy (26). The ability of a cell to persist drug treatment is not genetically predetermined (2, 27), but it is not clear if stable, nongenetic preexisting features drive this process. Shaffer and colleagues showed that in melanoma, rare cells in a population express resistance-promoting genes allowing survival during drug treatment, but upon expansion of these cells in the absence of drug most cells revert to the low-level expression observed in the original population (17). Barcoding studies of persister cells to chemotherapy (28–30) or HER2 inhibitors (16) showed no enrichment for specific clonal populations, indicating the ability to persist is a stochastic process that can potentially occur in any tumor cell. However, a recent study of osimertinib persisters (15) found that heritable hypophosphorylation of the IRS1 protein could enhance the ability of cells to survive drug treatment. Interestingly, Kurppa and colleagues found that DTPs generated via osimertinib monotherapy were enriched for particular barcoded clones in multiple parallel experiments; however, this enrichment was lost under the more stringent treatment regimen of an osimertinib–trametinib combination (22). Our study contributes to the knowledge in this field by showing that drug treatment does not select preexisting subclones that are fully resistant to osimertinib-induced apoptosis in a heritable manner, as cells that re-grow upon drug release show a strong apoptotic response when re-challenged with drug. However, this does not exclude the possibility that there exist heritable factors along a spectrum that cause pre-existing clones to have a higher probability of surviving drug treatment.

In this study, we show that while residual cells can avoid EGFR inhibitor–mediated cell death, they remain apoptotically competent, with several agents possessing the ability to push cells into activating the apoptotic cascade. We have shown that agents that re-activate either the intrinsic or extrinsic pathway can each inhibit the formation of osimertinib DTPs, as well as eliminate a significant number of DTPs once established. However, it was notable that the SMAC mimetic, AZD5582 was superior to BH3 mimetics when given as an up-front combination both *in vitro* and *in vivo*, preventing or delaying regrowth after removal of drug (Figs. 3D and 4). These data show that in addition to augmenting apoptosis, the AZD5582 combination can likely induce a state of senescence (Fig. 3E and F) which contributes to its anti-tumor effects. Moreover, combining AZD5582 and MCL1 inhibition in established DTPs augments BIM accumulation and can effectively eliminate nearly all persistent cells (Fig. 3G; Supplementary Fig. S3F).

Several studies have confirmed that the induction of BIM is critical to the execution of apoptotic death after EGFR-TKI treatment (5–7), and we corroborate these studies by showing that BIM-deleted cell lines form a significantly increased number of DTPs (Fig. 1E). Interestingly, there exists a germline BIM deletion polymorphism, whereby the gene lacks the BH3 domain, which appears to diminish the clinical response to EGFR-TKI treatment (31, 32). Despite the important role for BIM in osimertinib-driven apoptosis, we paradoxically found elevated levels of BIM protein in the DTP population (Fig. 1B and C). While it is well established that acute EGFR-TKI treatment elevates BIM by blocking ERK signaling (8), this link is at least partially uncoupled in DTPs, as they show a recovery of ERK activity yet have equal or greater levels of BIM protein (Fig. 1B). The antiapoptotic proteins BCL2, Bcl-xL, and MCL1 can each bind and inhibit BIM to prevent it from promoting Bak/Bax–driven Cytochrome C release (33), with different tumors showing distinct preferences for individual prosurvival BCL2 family proteins (34). In PC9 DTPs, it appears that both Bcl-xL and MCL1 can mediate this effect, as AZD5991, AZD4320, but not the Bcl-2–specific drug venetoclax, could enhance DTP cell death (Fig. 2A; Supplementary Fig. S2A–S2I). We also show evidence that surviving DTPs can switch from MCL1 to Bcl-xL, or vice versa, when challenged with the relevant BH3 mimetic drug, as switching between AZD5991 and AZD4320 combinations partway through the treatment phase eliminated a greater number of DTPs that a single combination maintained throughout (Fig. 2B, Supplementary Fig. S2E). Indeed, this phenomenon has been documented in AML cell lines, where increased expression and binding of MCL1 to BIM has been validated as a resistance mechanism to venetoclax (35, 36). Critically, it has been shown that sensitivity to BH3 mimetics does not depend on expression of target BCL2 family proteins (37), but rather is dependent on displacement of BH3 complexes (38). In keeping with this notion, we saw no significant changes in relevant antiapoptotic BCL2-family mRNA expression upon osimertinib treatment (Supplementary Fig. S6A–S6C). Interestingly, we observed a similar level of caspase activation with the osimertinib/AZD5991 combination in BIM knockout cells compared with control (Supplementary Fig. S2F), which led to significant inhibition of the DTP phenotype (Fig. 2D). This indicates that MCL1 regulates a BIM-independent apoptotic mechanism after EGFR inhibitor treatment. One candidate for the effector of this phenomenon is BID, another proapoptotic BH3-only protein that has been shown to bind MCL1 (39).

Cell death is not limited to apoptosis, and numerous other mechanisms have been documented which result in tumor cell killing. Recently, there has been an increased interest in ferroptosis, an iron-dependent process whereby toxic oxidated phospholipids trigger a rapid and catastrophic cascade leading to cellular death (40). DTPs appear to be particularly sensitive to inhibition of GPX4 (41), the master regulator of ferroptosis. To date, no GPX4 inhibitors have been approved for use in the clinic, and any such inhibitor may carry significant safety liabilities, as it has been shown that mice with a specific deletion of GPX4 in the kidney display acute renal failure (42). The combinations of osimertinib with either AZD5991 or AZD5582 were well-tolerated in mice, and the former has entered clinical trials for patients with hematologic malignancies, either alone or in combination with venetoclax. In this study, we have shown that AZD5991 and AZD5582 monotherapy treatment can induce apoptosis in established DTPs (Figs. 2A and 3D), and thus alternate scheduling of these drugs may provide clinical benefit while maintaining a favorable safety profile. The fact that DTP cells are primed for apoptosis by maintaining high levels of BIM means that lower doses of BH3 mimetics would be required for efficacy (43), again mitigating any potential toxicity. We also note that advances in quantifying the *EGFR* mutation in circulating tumor DNA after osimertinib treatment has the potential to quickly identify likely poor responding patients (44), who might be candidates for more aggressive treatment regimens such as those proposed in this study.

A feature of drug tolerance is the initiation of a distinct transcriptional program that supports cell survival in the absence of proliferation. Previous studies have identified several phenotypic outputs of this altered transcription, including epithelial-to-mesenchymal transition (EMT), the acquisition of a stem-like cellular state (45), and survival signaling driven by YAP-TEAD gene regulation. Indeed, it is likely that each of these pathways are invoked in a coordinated manner to drive drug tolerance. A key output of enhanced YAP-TEAD activity in DTPs is the repression of the proapoptotic gene *BMF* and the attenuation the apoptotic response (22). The interdependence of these transcriptional programs is highlighted by the finding that this process is dependent on Slug, a key transcription factor that mediates EMT. Interestingly, while not a member of the canonical YAP-TEAD signature, it has been shown that *BIRC3* is positively regulated by this pathway certain contexts (46). We postulate that this could further promote YAP-mediated cell survival in drug-tolerant persisters beyond the previously described effects on *BMF*.

In contrast, our study shows that once osimertinib resistance (i.e., the ability to actively proliferate in the presence of drug) has been established, neither BH3 mimetics, SMAC mimetics, nor the inhibition of YAP-TEAD signaling are able to sensitize cells to EGFR inhibition (Supplementary Fig. S5F and S5G). However, when combined with agents which target the driver of resistance, each of these classes of agents further sensitized resistant cells to osimertinib in proliferation assays. In the case of the YAP-TEAD, we postulate that this pathway primarily regulates cell survival only when the driver oncogene is inhibited, and in resistant cells pathways that bypass EGFR signaling also bypasses the requirement for YAP-TEAD. Recent reports have established that there is significant cross-talk between the YAP and MAPK pathways (47, 48) which converge on regulating TEAD activity. This suggests that in cases of EGFR-TKI resistance driven by MAPK alterations, targeting a common node in these two pathways could be an attractive strategy for delaying or preventing relapse in the clinic.

In summary, we have shown that osimertinib drug-tolerant cells, which by definition are refractory to cell death, maintain apoptotic competence that can be triggered by agents acting upon multiple nodes of this cell death cascade. However once resistance has been established, maximizing apoptosis with these same agents, while still possible, is more challenging due to requirement for inhibiting a diverse set of driver oncogenes while maintaining the original EGFR-TKI treatment. Taken together, these data indicate that intervening with combinations that maximize the induction of apoptosis, either up-front or at the minimal residual disease stage, could be effective at delaying or even preventing the development of acquired resistance, and suggest novel treatment strategies that warrant further exploration.

## Supplementary Material

Supplementary Tables S1-S2, Figures S1-S6 Supplementary Table 1. Cell lines used. Supplementary Table 2. Antibodies used. Supplementary Figure S1. Osimertinib drug tolerant cells re-aquire apoptotic capacity after drug holiday. Supplementary Figure S2. BH3 mimetics can trigger apoptosis in established DTPs. Supplementary Figure S3. DTPs upregulate cIAP proteins and are sensitive to SMAC mimetic treatment. Supplementary Figure S4. SMAC mimetics inhibit DTP growth in vivo. Supplementary Figure S5. BH3 and SMAC mimetics enhance apoptosis induced by agents targeting resistance drivers. Supplementary Figure S6. Measuring transcript levels for anti-apoptotic genes in osimertinib DTPs.

## References

[1] Jänne PA , Yang JCH, Kim DW, Planchard D, Ohe Y, Ramalingam SS, . AZD9291 in EGFR inhibitor-resistant non-small-cell lung cancer. N Engl J Med 2015;372:1689–99. 25923549 10.1056/NEJMoa1411817

[2] Sharma SV , Lee DY, Li B, Quinlan MP, Takahashi F, Maheswaran S, . A chromatin-mediated reversible drug-tolerant state in cancer cell subpopulations. Cell 2010;141:69–80. 20371346 10.1016/j.cell.2010.02.027 PMC2851638

[3] Ramirez M , Rajaram S, Steininger RJ, Osipchuk D, Roth MA, Morinishi LS, . Diverse drug-resistance mechanisms can emerge from drug-tolerant cancer persister cells. Nat Commun 2016;7:10690. 26891683 10.1038/ncomms10690 PMC4762880

[4] Guler GD , Tindell CA, Pitti R, Wilson C, Nichols K, KaiWai Cheung T, . Repression of stress-induced LINE-1 expression protects cancer cell subpopulations from lethal drug exposure. Cancer Cell 2017;32:221–37e13. 28781121 10.1016/j.ccell.2017.07.002

[5] Costa DB , Halmos B, Kumar A, Schumer ST, Huberman MS, Boggon TJ, . BIM mediates EGFR tyrosine kinase inhibitor-induced apoptosis in lung cancers with oncogenic EGFR mutations. PLoS Med 2007;4:1669–79; discussion 1680. 17973572 10.1371/journal.pmed.0040315 PMC2043012

[6] Deng J , Shimamura T, Perera S, Carlson NE, Cai D, Shapiro GI, . Proapoptotic BH3-only BCL-2 family protein BIM connects death signaling from epidermal growth factor receptor inhibition to the mitochondrion. Cancer Res 2007;67:11867–75. 18089817 10.1158/0008-5472.CAN-07-1961

[7] Cragg MS , Kuroda J, Puthalakath H, Huang DCS, Strasser A. Gefitinib-induced killing of NSCLC cell lines expressing mutant EGFR requires BIM and can be enhanced by BH3 mimetics. PLoS Med 2007;4:1681–89; discussion 1690. 17973573 10.1371/journal.pmed.0040316 PMC2043013

[8] Shi P , Oh YT, Deng L, Zhang G, Qian G, Zhang S, . Overcoming acquired resistance to AZD9291, a third-generation EGFR inhibitor, through modulation of MEK/ERK-dependent Bim and Mcl-1 degradation. Clin Cancer Res 2017;23:6567–79. 28765329 10.1158/1078-0432.CCR-17-1574 PMC5668147

[9] Falschlehner C , Emmerich CH, Gerlach B, Walczak H. TRAIL signalling: decisions between life and death. Int J Biochem Cell Biol 2007;39:1462–75. 17403612 10.1016/j.biocel.2007.02.007

[10] Rathore R , McCallum JE, Varghese E, Florea AM, Büsselberg D. Overcoming chemotherapy drug resistance by targeting inhibitors of apoptosis proteins (IAPs). Apoptosis 2017;22:898–919. 28424988 10.1007/s10495-017-1375-1 PMC5486846

[11] Boddu P , Carter BZ, Verstovsek S, Pemmaraju N. SMAC mimetics as potential cancer therapeutics in myeloid malignancies. Br J Haematol 2019;185:219–31. 30836448 10.1111/bjh.15829

[12] Eberlein CA , Stetson D, Markovets AA, Al-Kadhimi KJ, Lai Z, Fisher PR, . Acquired resistance to the mutant-selective EGFR inhibitor AZD9291 is associated with increased dependence on RAS signaling in preclinical models. Cancer Res 2015;75:2489–500. 25870145 10.1158/0008-5472.CAN-14-3167 PMC4605607

[13] Vaclova T , Grazini U, Ward L, O'Neill D, Markovets A, Huang X, . Clinical impact of subclonal EGFR T790M mutations in advanced-stage EGFR-mutant non-small-cell lung cancers. Nat Commun 2021;12:1780. 33741979 10.1038/s41467-021-22057-8 PMC7979775

[14] Cidado J , Boiko S, Proia T, Ferguson D, Criscione SW, San Martin M, . AZD4573 is a highly selective CDK9 inhibitor that suppresses MCL-1 and induces apoptosis in hematologic cancer cells. Clin Cancer Res 2020;26:922–34. 31699827 10.1158/1078-0432.CCR-19-1853

[15] Jacob Berger A , Gigi E, Kupershmidt L, Meir Z, Gavert N, Zwang Y, . IRS1 phosphorylation underlies the non-stochastic probability of cancer cells to persist during EGFR inhibition therapy. Nat Cancer 2021;2:1055–70. 35121883 10.1038/s43018-021-00261-1

[16] Chang CA , Jen J, Jiang S, Sayad A, Mer AS, Brown KR, . Ontogeny and vulnerabilities of drug-tolerant persisters in HER2+ breast cancer. Cancer Discov 2022;12:1022–45. 34911733 10.1158/2159-8290.CD-20-1265 PMC8983469

[17] Shaffer SM , Dunagin MC, Torborg SR, Torre EA, Emert B, Krepler C, . Rare cell variability and drug-induced reprogramming as a mode of cancer drug resistance. Nature 2017;546:431–5. 28607484 10.1038/nature22794 PMC5542814

[18] Ley R , Balmanno K, Hadfield K, Weston C, Cook SJ. Activation of the ERK1/2 signaling pathway promotes phosphorylation and proteasome-dependent degradation of the BH3-only protein, Bim. J Biol Chem 2003;278:18811–6. 12646560 10.1074/jbc.M301010200

[19] Willis SN , Fletcher JI, Kaufmann T, van Delft MF, Chen L, Czabotar PE, . Apoptosis initiated when BH3 ligands engage multiple Bcl-2 homologs, not Bax or Bak. Science 2007;315:856–9. 17289999 10.1126/science.1133289

[20] Tron AE , Belmonte MA, Adam A, Aquila BM, Boise LH, Chiarparin E, . Discovery of Mcl-1-specific inhibitor AZD5991 and preclinical activity in multiple myeloma and acute myeloid leukemia. Nat Commun 2018;9:5341. 30559424 10.1038/s41467-018-07551-w PMC6297231

[21] Balachander SB , Criscione SW, Byth KF, Cidado J, Adam A, Lewis P, . AZD4320, a dual inhibitor of Bcl-2 and Bcl-x_L_, induces tumor regression in hematologic cancer models without dose-limiting thrombocytopenia. Clin Cancer Res 2020;26:6535–49. 32988967 10.1158/1078-0432.CCR-20-0863

[22] Kurppa KJ , Liu Y, To C, Zhang T, Fan M, Vajdi A, . Treatment-induced tumor dormancy through YAP-mediated transcriptional reprogramming of the apoptotic pathway. Cancer Cell 2020;37:104–22. 31935369 10.1016/j.ccell.2019.12.006 PMC7146079

[23] Kaneda A , Seike T, Danjo T, Nakajima T, Otsubo N, Yamaguchi D, . The novel potent TEAD inhibitor, K-975, inhibits YAP1/TAZ-TEAD protein-protein interactions and exerts an anti-tumor effect on malignant pleural mesothelioma. Am J Cancer Res 2020;10:4399–415. 33415007 PMC7783735

[24] Hennessy EJ , Adam A, Aquila BM, Castriotta LM, Cook D, Hattersley M, . Discovery of a novel class of dimeric Smac mimetics as potent IAP antagonists resulting in a clinical candidate for the treatment of cancer (AZD5582). J Med Chem 2013;56:9897–919. 24320998 10.1021/jm401075x

[25] Tricker EM , Xu C, Uddin S, Capelletti M, Ercan D, Ogino A, . Combined EGFR/MEK inhibition prevents the emergence of resistance in EGFR-mutant lung cancer. Cancer Discov 2015;5:960–71. 26036643 10.1158/2159-8290.CD-15-0063 PMC4824006

[26] Hata AN , Niederst MJ, Archibald HL, Gomez-Caraballo M, Siddiqui FM, Mulvey HE, . Tumor cells can follow distinct evolutionary paths to become resistant to epidermal growth factor receptor inhibition. Nat Med 2016;22:262–9. 26828195 10.1038/nm.4040 PMC4900892

[27] Recasens A , Munoz L. Targeting cancer cell dormancy. Trends Pharmacol Sci 2019;40:128–41. 30612715 10.1016/j.tips.2018.12.004

[28] Rehman SK , Haynes J, Collignon E, Brown KR, Wang Y, Nixon AML, . Colorectal cancer cells enter a diapause-like DTP state to survive chemotherapy. Cell 2021;184:226–42. 33417860 10.1016/j.cell.2020.11.018 PMC8437243

[29] Dhimolea E , de Matos Simoes R, Kansara D, Al'Khafaji A, Bouyssou J, Weng X, . An embryonic diapause-like adaptation with suppressed Myc activity enables tumor treatment persistence. Cancer Cell 2021;39:240–56. 33417832 10.1016/j.ccell.2020.12.002 PMC8670073

[30] Duy C , Li M, Teater M, Meydan C, Garrett-Bakelman FE, Lee TC, . Chemotherapy induces senescence-like resilient cells capable of initiating AML recurrence. Cancer Discov 2021;11:1542–61. 33500244 10.1158/2159-8290.CD-20-1375 PMC8178167

[31] Su W , Zhang X, Cai X, Peng M, Wang F, Wang Y. BIM deletion polymorphism predicts poor response to EGFR-TKIs in nonsmall cell lung cancer: An updated meta-analysis. Medicine (Baltimore) 2019;98:e14568. 30855441 10.1097/MD.0000000000014568 PMC6417537

[32] Ng KP , Hillmer AM, Chuah CTH, Juan WC, Ko TK, Teo AS, . A common BIM deletion polymorphism mediates intrinsic resistance and inferior responses to tyrosine kinase inhibitors in cancer. Nat Med 2012;18:521–8. 22426421 10.1038/nm.2713

[33] Juin P , Geneste O, Gautier F, Depil S, Campone M. Decoding and unlocking the BCL-2 dependency of cancer cells. Nat Rev Cancer 2013;13:455–65. 23783119 10.1038/nrc3538

[34] Singh R , Letai A, Sarosiek K. Regulation of apoptosis in health and disease: the balancing act of BCL-2 family proteins. Nat Rev Mol Cell Biol 2019;20:175–93. 30655609 10.1038/s41580-018-0089-8 PMC7325303

[35] Niu X , Zhao J, Ma J, Xie C, Edwards H, Wang G, . Binding of released Bim to Mcl-1 is a mechanism of intrinsic resistance to ABT-199 which can be overcome by combination with daunorubicin or cytarabine in AML cells. Clin Cancer Res 2016;22:4440–51. 27103402 10.1158/1078-0432.CCR-15-3057 PMC5010519

[36] Yecies D , Carlson NE, Deng J, Letai A. Acquired resistance to ABT-737 in lymphoma cells that up-regulate MCL-1 and BFL-1. Blood 2010;115:3304–13. 20197552 10.1182/blood-2009-07-233304 PMC2858493

[37] Liu Q , Osterlund EJ, Chi X, Pogmore J, Leber B, Andrews DW. Bim escapes displacement by BH3-mimetic anti-cancer drugs by double-bolt locking both Bcl-XL and Bcl-2. Elife 2019;8:e37689. 30860026 10.7554/eLife.37689 PMC6414199

[38] Osterlund EJ , Hirmiz N, Pemberton JM, Nougarède A, Liu Q, Leber B, . Efficacy and specificity of inhibitors of BCL-2 family protein interactions assessed by affinity measurements in live cells. Sci Adv 2022;8:eabm7375. 35442739 10.1126/sciadv.abm7375 PMC9020777

[39] Clohessy JG , Zhuang J, de Boer J, Gil-Gómez G, Brady HJM. Mcl-1 interacts with truncated Bid and inhibits its induction of cytochrome c release and its role in receptor-mediated apoptosis. J Biol Chem 2006;281:5750–9. 16380381 10.1074/jbc.M505688200

[40] Dixon SJ , Lemberg KM, Lamprecht MR, Skouta R, Zaitsev EM, Gleason CE, . Ferroptosis: an iron-dependent form of nonapoptotic cell death. Cell 2012;149:1060–72. 22632970 10.1016/j.cell.2012.03.042 PMC3367386

[41] Hangauer MJ , Viswanathan VS, Ryan MJ, Bole D, Eaton JK, Matov A, . Drug-tolerant persister cancer cells are vulnerable to GPX4 inhibition. Nature 2017;551:247–50. 29088702 10.1038/nature24297 PMC5933935

[42] Friedmann Angeli JP , Schneider M, Proneth B, Tyurina YY, Tyurin VA, Hammond VJ, . Inactivation of the ferroptosis regulator Gpx4 triggers acute renal failure in mice. Nat Cell Biol 2014;16:1180–91. 25402683 10.1038/ncb3064 PMC4894846

[43] Bolomsky A , Vogler M, Köse MC, Heckman CA, Ehx G, Ludwig H, . MCL-1 inhibitors, fast-lane development of a new class of anti-cancer agents. J Hematol Oncol 2020;13:173. 33308268 10.1186/s13045-020-01007-9 PMC7731749

[44] Buder A , Hochmair MJ, Setinek U, Pirker R, Filipits M. *EGFR* mutation tracking predicts survival in advanced *EGFR*-mutated non-small cell lung cancer patients treated with osimertinib. Transl Lung Cancer Res 2020;9:239–45. 32420063 10.21037/tlcr.2020.03.02 PMC7225165

[45] Morales Torres C , Wu MY, Hobor S, Wainwright EN, Martin MJ, Patel H, . Selective inhibition of cancer cell self-renewal through a Quisinostat-histone H1.0 axis. Nat Commun 2020;11:1792. 32286289 10.1038/s41467-020-15615-z PMC7156485

[46] Codelia VA , Sun G, Irvine KD. Regulation of YAP by mechanical strain through Jnk and Hippo signaling. Curr Biol 2014;24:2012–7. 25127217 10.1016/j.cub.2014.07.034 PMC4160395

[47] Pham TH , Hagenbeek TJ, Lee HJ, Li J, Rose CM, Lin E, . Machine-learning and chemicogenomics approach defines and predicts cross-talk of Hippo and MAPK pathways. Cancer Discov 2021;11:778–93. 33208393 10.1158/2159-8290.CD-20-0706

[48] Garcia-Rendueles MER , Ricarte-Filho JC, Untch BR, Landa I, Knauf JA, Voza F, . NF2 Loss promotes oncogenic RAS-induced thyroid cancers via YAP-dependent transactivation of RAS proteins and sensitizes them to MEK inhibition. Cancer Discov 2015;5:1178–93. 26359368 10.1158/2159-8290.CD-15-0330 PMC4642441

