# Supplementary material for: Pharmaceutical Reactivation of Attenuated Apoptotic Pathways Leads to Elimination of Osimertinib Drug-Tolerant Cells
Source: Cancer Res Commun. 2022 Oct 31;2(10):1312–25. doi: 10.1158/2767-9764.CRC-22-0066 (PMC10035388; doi:10.1158/2767-9764.CRC-22-0066)
Supplement: Supplementary Tables S1-S2, Figures S1-S6 — Supplementary Table 1. Cell lines used. Supplementary Table 2. Antibodies used. Supplementary Figure S1. Osimertinib drug tolerant cells re-aquire apoptotic capacity after drug holiday. Supplementary Figure S2. BH3 mimetics can trigger apoptosis in established DTPs. Supplementary Figure S3. DTPs upregulate cIAP proteins and are sensitive to SMAC mimetic treatment. Supplementary Figure S4. SMAC mimetics inhibit DTP growth in vivo. Supplementary Figure S5. BH3 and SMAC mimetics enhance apoptosis induced by agents targeting resistance drivers. Supplementary Figure S6. Measuring transcript levels for anti-apoptotic genes in osimertinib DTPs. [file crc-22-0066-s01.docx]

**SUPPLEMENTAL MATERIAL**

**Supplementary Table 1. Cell lines used.**

| **Cell Line** | **Source** | **Date Obtained** | **EGFR Mutation Status** |
| --- | --- | --- | --- |
| HCC2935 | ATCC | 2015 | Ex.19 Del |
| HCC4006 | ATCC | 2011 | Ex.19 Del |
| HCC827 | ATCC | 2012 | Ex.19 Del |
| HCC827-GR | In-house | 2017 | Ex.19 Del |
| II-18 | RCB | 2018 | L858R |
| NCI-H1975 | ATCC | 2004 | L858R; T790M |
| NCI-H1975 AZDR1 | In-house | 2013 | L858R; T790M |
| NCI-H1975 AZDR2 | In-house | 2013 | Ex.19 Del |
| PC9 | ECACC | 2006 | Ex.19 Del |
| PC9 AZDR1 | In-house | 2014 | Ex.19 Del |
| PC9 AZDR2 | In-house | 2014 | Ex.19 Del |
| PC9 AZDR3 | In-house | 2014 | Ex.19 Del |
| PC9 AZDR4 | In-house | 2014 | Ex.19 Del |
| PC9 NTC | In-house | 2019 | Ex.19 Del |
| PC9 sgBIM-1 | In-house | 2019 | Ex.19 Del |
| PC9 sgBIM-2 | In-house | 2019 | Ex.19 Del |
| PC9 (PIK3CA H1047R) | In-house | 2019 | Ex.19 Del |

**Supplementary Table 2. Antibodies used.**

| **Antibody Target** | **Source** | **Catalogue Number** | **RRID** | **Species** | **Dilution** |
| --- | --- | --- | --- | --- | --- |
| β-actin | Sigma-Aldrich | A2228 | AB_476679 | Mouse | 1:10,000 |
| BIM | Cell Signaling | 2933 | AB_1030947 | Rabbit | 1:1000 |
| cIAP1 | Cell Signaling | 7065 | AB_10890862 | Rabbit | 1:1000 |
| cIAP2 | Cell Signaling | 3130 | AB_10693298 | Rabbit | 1:1000 |
| Phospho-EGFR | Cell Signaling | 2234 | AB_331701 | Rabbit | 1:1000 |
| EGFR | Cell Signaling | 4267 | AB_2246311 | Rabbit | 1:1000 |
| Phospho-ERK | Cell Signaling | 9101 | AB_331646 | Rabbit | 1:2000 |
| ERK | Cell Signaling | 9102 | AB_330744 | Rabbit | 1:2000 |
| GAPDH | Cell Signaling | 5174 | AB_10622025 | Rabbit | 1:10,000 |
| Phospho-MET | Cell Signaling | 3077 | AB_2143884 | Rabbit | 1:1000 |
| MET | Cell Signaling | 8198 | AB_10858224 | Rabbit | 1:1000 |
| Phospho-S6 | Cell Signaling | 4858 | AB_916156 | Rabbit | 1:2000 |


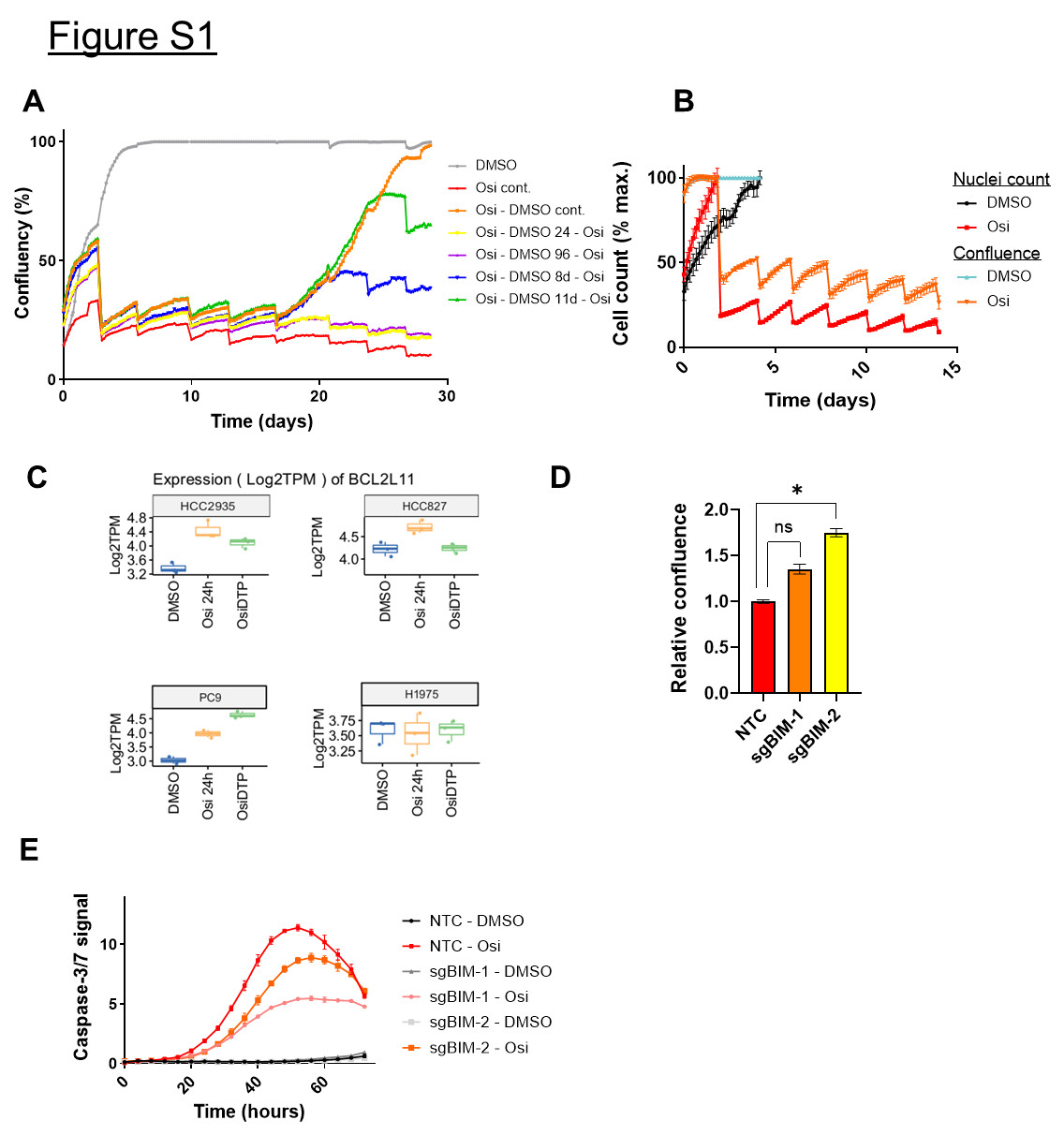


**Figure S1: Osimertinib drug tolerant cells re-aquire apoptotic capacity after drug holiday. A)** Cell confluence in PC9 cells treated with osimertinib for 10 days to generate DTPs, followed by varying periods of drug-free media prior to rechallenge with osimertinib (corresponding to Figure 1A) **B)** Comparison of confluence vs. nuclear count (Incucyte imaging platform) for PC9 cells treated with or without 500 nM osimertinib. Data are presented as relative to maximal confluence/nuclei count for the experiment. **C)** mRNA expression of *BCLC2L11* transcripts taken from RNAseq dataset in panel of EGFRm cell lines treated acutely (24h) or chronically (DTP; 21d) with 500 nM osimertinib. **D)** AUC of confluence plots for the indicated cell lines from the data presented in Fig. 1E. (*) p<0.05, two-tailed t-test. **E)** Caspase-3/7 activity in BIM-deleted PC9 cells treated acutely with 160 nM osimertinib, compared to control cells (NTC).

**
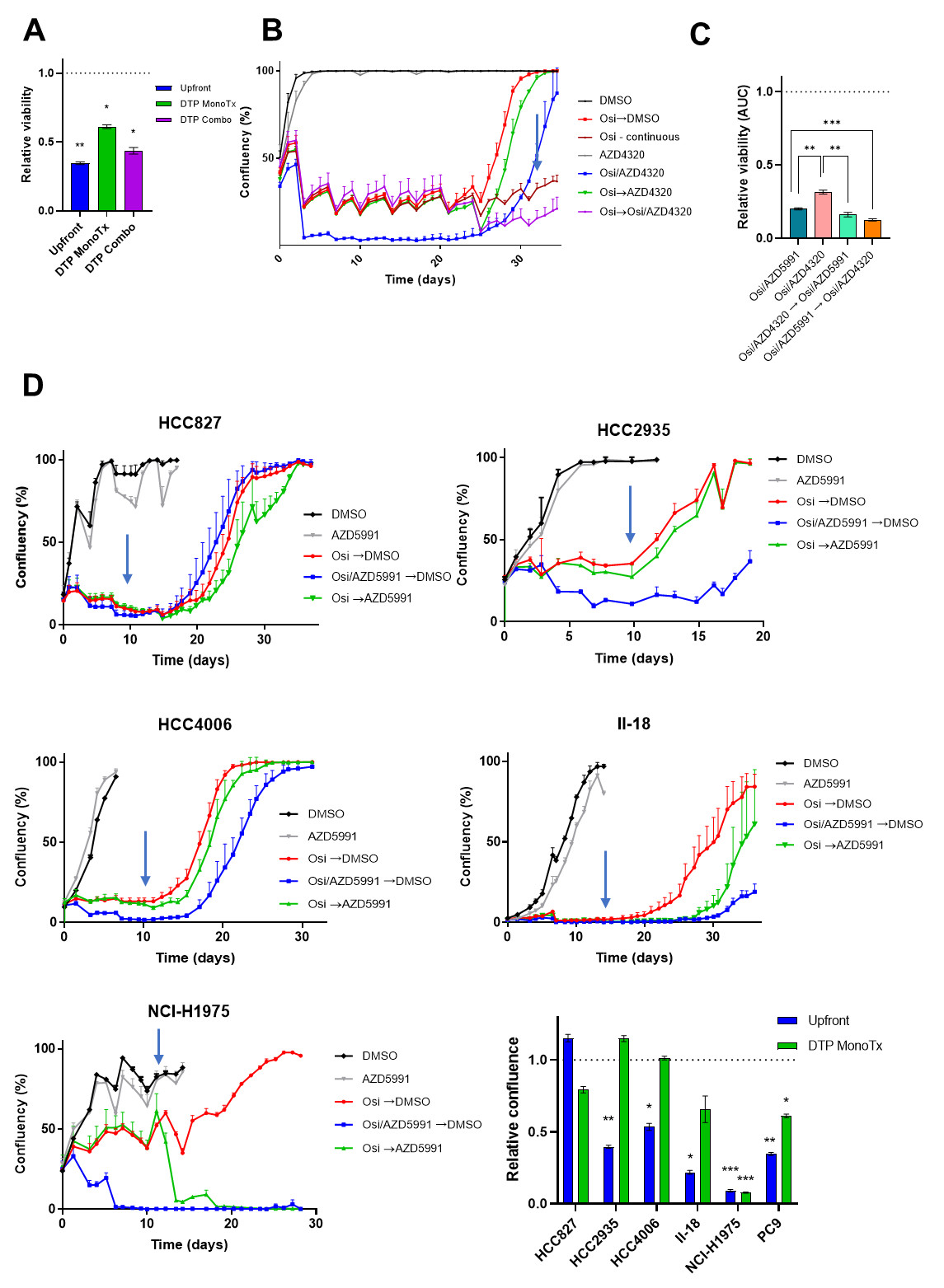
**

**
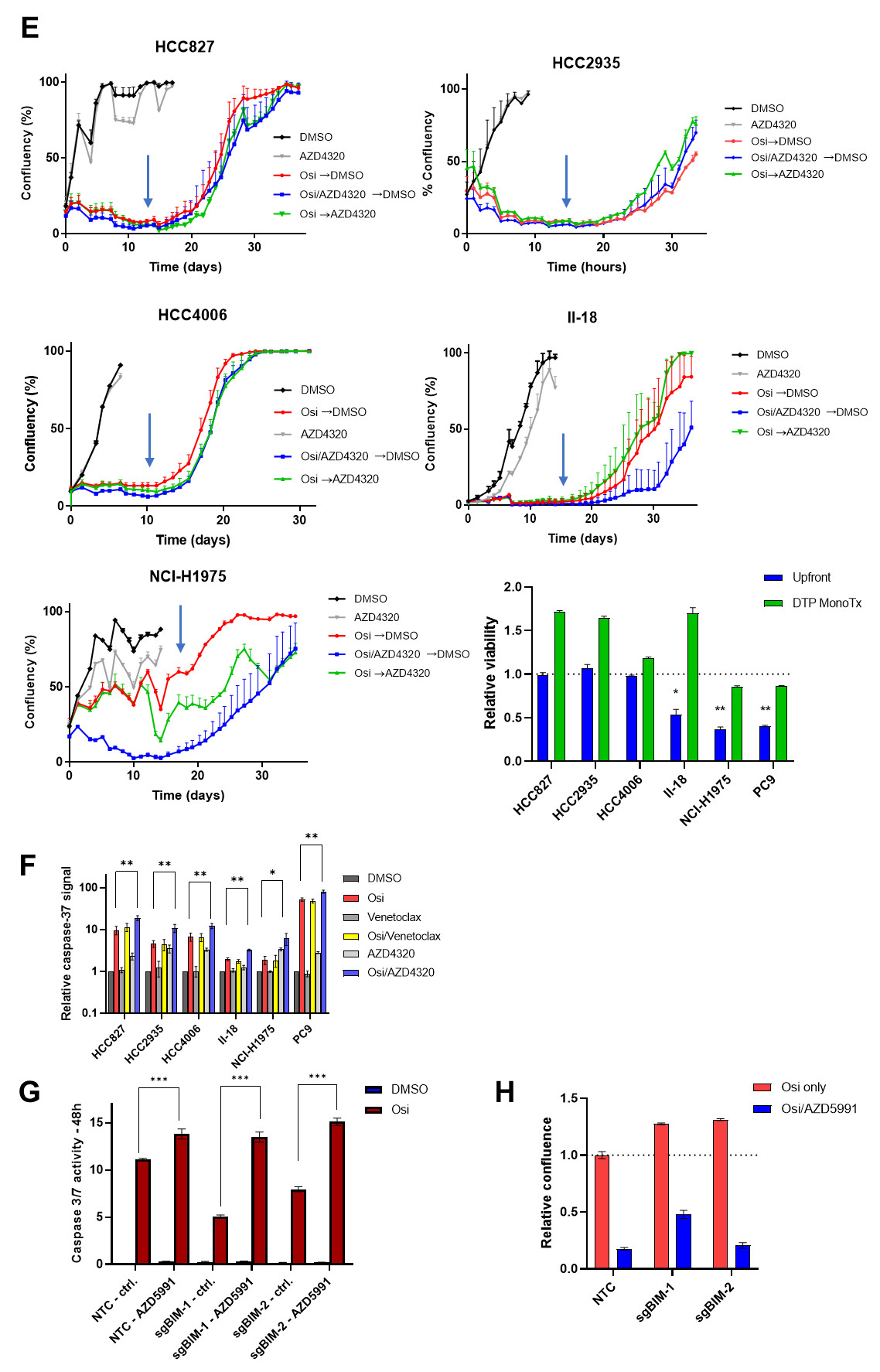
**

**
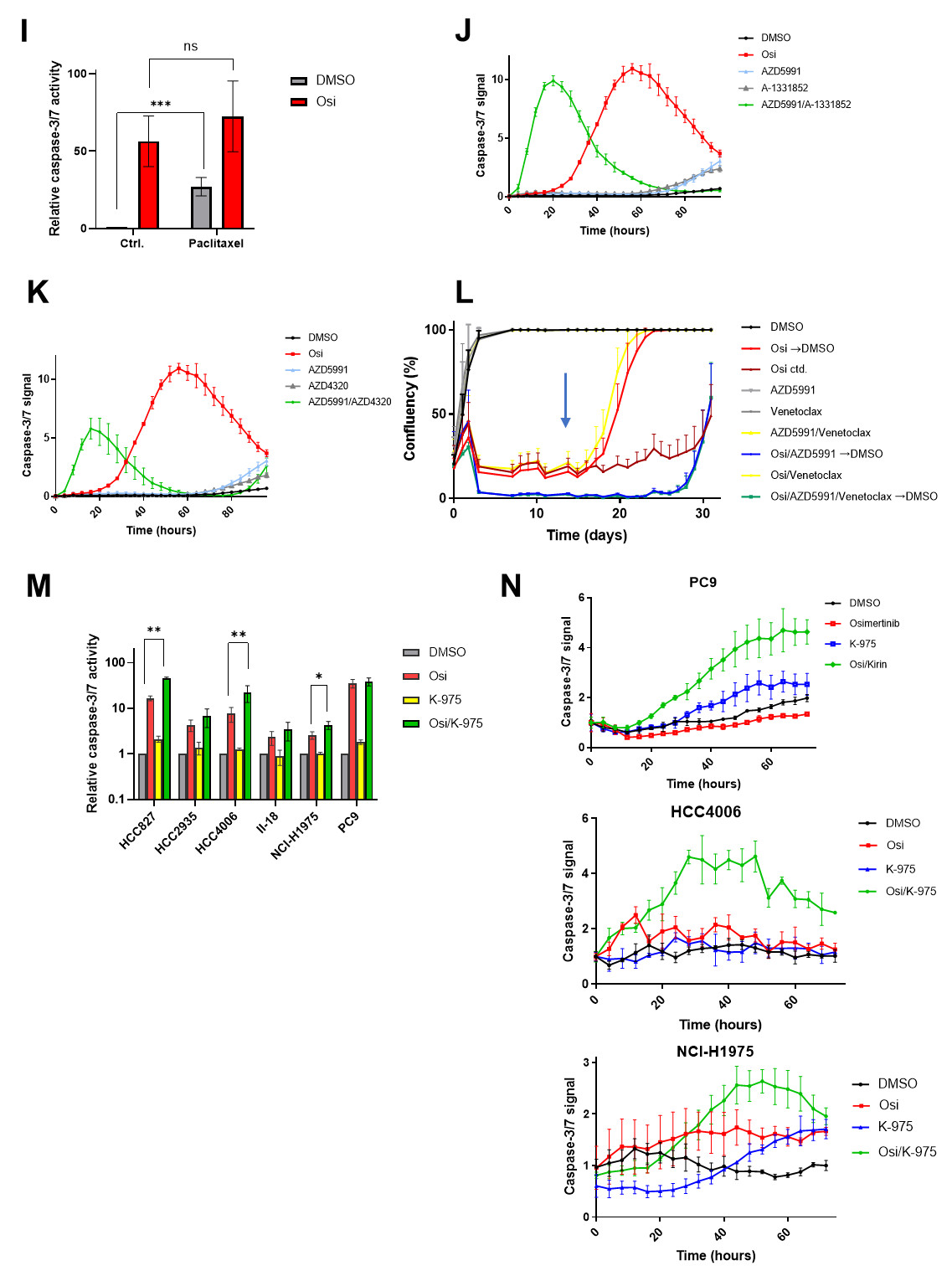
**

**Figure S2: BH3 mimetics can trigger apoptosis in established DTPs.** **A)** AUC of confluence plots for PC9 AZD5991 DTPs compared to DTP monotherapy, corresponding to the data presented in Fig. 2A. **B)** DTP assay in PC9 cells measuring effect of AZD4320 (300 nM) in upfront combination (blue) and DTP monotherapy (green) vs. osimertinib (500 nM) monotherapy controls (red). Arrow indicates change in dosing regimen. **C)** AUC of confluence plots for PC9 combination treatment DTPs, corresponding to the data presented in Fig. 2B. **D)** DTP assay in the indicated cell lines measuring effect of AZD5991 (300 nM) in upfront combination (blue) and DTP monotherapy (green) vs. osimertinib monotherapy controls (red). Arrow indicates change in dosing regimen. Bar chart summarises AUC for each of the AZD5991-containing treatments compared to osimertinib monotherapy. **E)** DTP assay in the indicated cell lines measuring effect of AZD4320 (300 nM) in upfront combination (blue) and DTP monotherapy (green) vs. osimertinib monotherapy controls (red). Arrow indicates change in dosing regimen. Bar chart summarises AUC for each of the AZD5991-containing treatments compared to osimertinib monotherapy. **F)** Caspase-3/7 activity in a panel of EGFRm cells treated for 72h with osimertinib, the indicated drugs (osimertinib 160 nM; venetoclax 300 nM; AZD4320 300 nM), compared to control. Values represent AUC of caspase-3/7 signal for the 72h duration of the experiment. **E)** Caspase-3/7 activity in BIM-deleted PC9 cells treated for 48h (single time point) with osimertinib (160 nM), AZD5991 (300 nM) or their combination compared to control (NTC). **G)** Relative caspase-3/7 activity in a control vs. BIM knockout PC9 cells at a single time point (48h) after treatment with the indicated compounds. **H)** AUC of confluence plots for PC9 control or BIM-knockout DTPs treated with osimertinib monotherapy or its combination with AZD5991.**I)** Caspase-3/7 activity in a panel of EGFRm cells treated for 72h with osimertinib (160 nM), paclitaxel (30 nM) or their combination, compared to control. Values represent AUC of caspase-3/7 signal for the duration of the experiment. **J/K** Caspase-3/7 activity in PC9 cells treated with the indicated drugs (osimertinib 160 nM; AZD5991 300 nM; A-1331852 100 nM; AZD4320 300 nM). **M)** Caspase-3/7 activity in a panel of EGFRm cells treated for 72h with drugs as indicated (osimertinib 160 nM; K-975 100 nM), compared to control. Values represent AUC of caspase-3/7 signal for the 72h duration of the experiment. N) Caspase-3-7 activity in established DTPs from PC9, HCC4006 and NCI-H1975 cells, treated for 72h with the indicated compounds (osimertinib 500 nM; K-975 100 nM).

Mean ± SD are shown in all plots. Two-tailed t-tests were used for statistical analyses. ***p<0.001, **p<0.01, *p<0.05, NS = not significant.

**
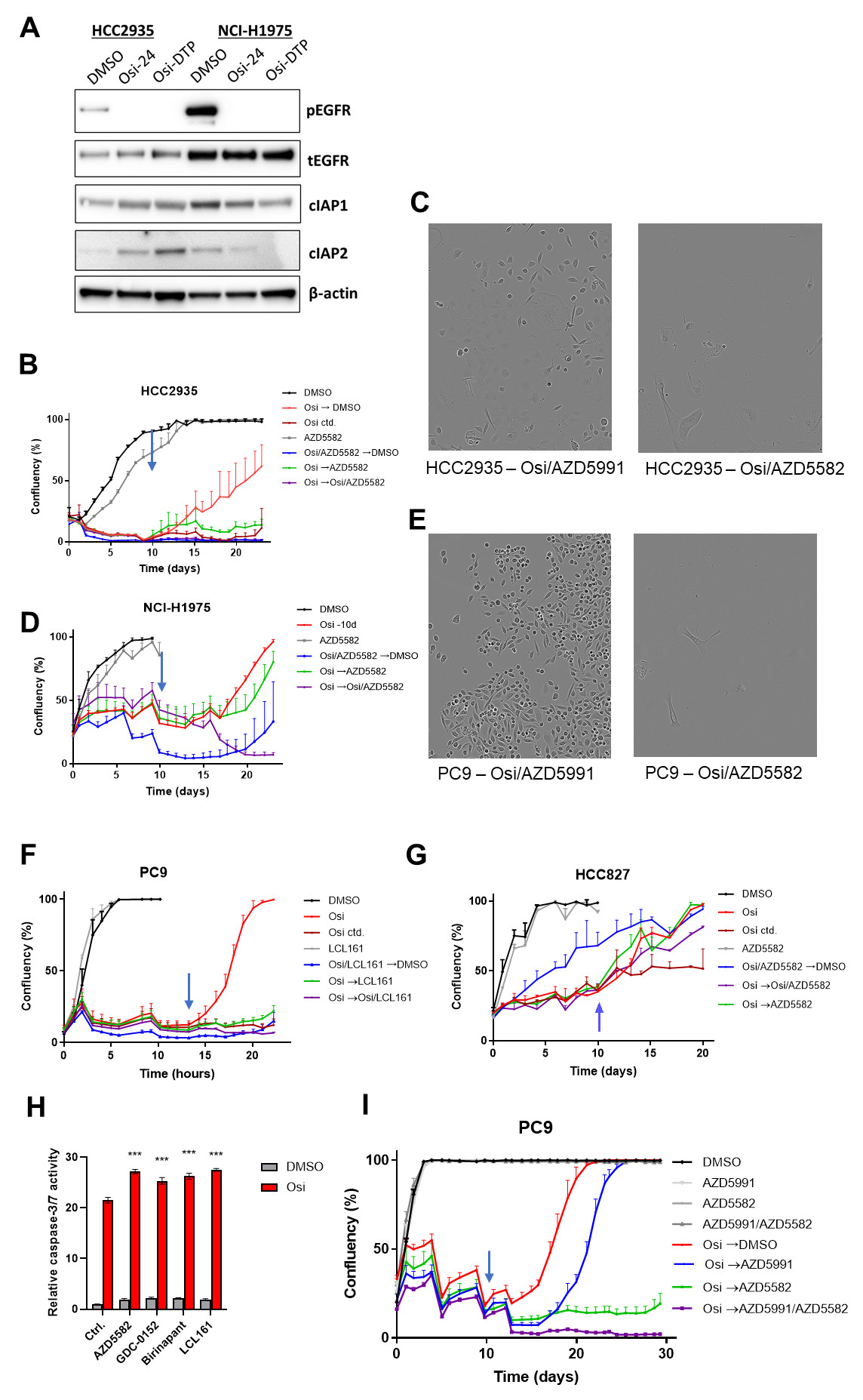
**

**Figure S3: DTPs upregulate cIAP proteins and are sensitive to SMAC mimetic treatment. A)** Western blot showing relative expression of the indicated proteins after acute (24h) osimertinib treatment compared to DTPs. **B)** HCC2935 DTP assay measuring effect of AZD5582 in upfront combination (blue), DTP monotherapy (green) and DTP combination (violet) vs. osimertinib monotherapy (100 nM) controls (red). Arrow indicates time of change in dosing regimen. **C)** Images taken of HCC2935 cells from the experiment in Fig. S3B treated for 10 days with either AZD5991 (300 nM) or AZD5582 (100 nM) in combination with osimertinib, followed by 7 days of culture in drug-free media. **D)** NCI-H1975 DTP assay measuring effect of AZD5582 (100 nM) in upfront combination (blue), DTP monotherapy (green) and DTP combination (violet) vs. osimertinib monotherapy (500 nM) controls (red). Arrow indicates time of change in dosing regimen. **E)** Images taken of PC9 cells from the experiment in Fig. 3D treated for 10 days with either AZD5991 (300 nM) or AZD5582 (100 nM) in combination with osimertinib, followed by 7 days of culture in drug-free media. **F)** PC9 DTP assay measuring effect of LCL161 (100 nM) in upfront combination (blue), DTP monotherapy (green) and DTP combination (violet) vs. osimertinib monotherapy (500 nM) controls (red). Arrow indicates time of change in dosing regimen. **G)** HCC827 DTP assay measuring effect of AZD5582 (100 nM) in upfront combination (blue), DTP monotherapy (green) and DTP combination (violet) vs. osimertinib monotherapy (500 nM) controls (red). Arrow indicates time of change in dosing regimen. **H)** Caspase-3/7 activity in PC9 cells treated for 72h with osimertinib (160 nM) with or without the combination of the indicated SMAC mimetic (all at 100 nM) compared to untreated control. Values represent AUC of caspase-3/7 signal for the duration of the experiment. **I)** PC9 DTP assay measuring effect of the AZD5991 monotherapy (blue) AZD5582 (green) or their combination (violet) in established DTPs compared to drug-free controls (red). Arrow indicates time of change in dosing regimen.

Mean ± SD are shown in all plots. Two-tailed t-tests were used for statistical analyses. ***p<0.001, **p<0.01, *p<0.05, NS = not significant.

**
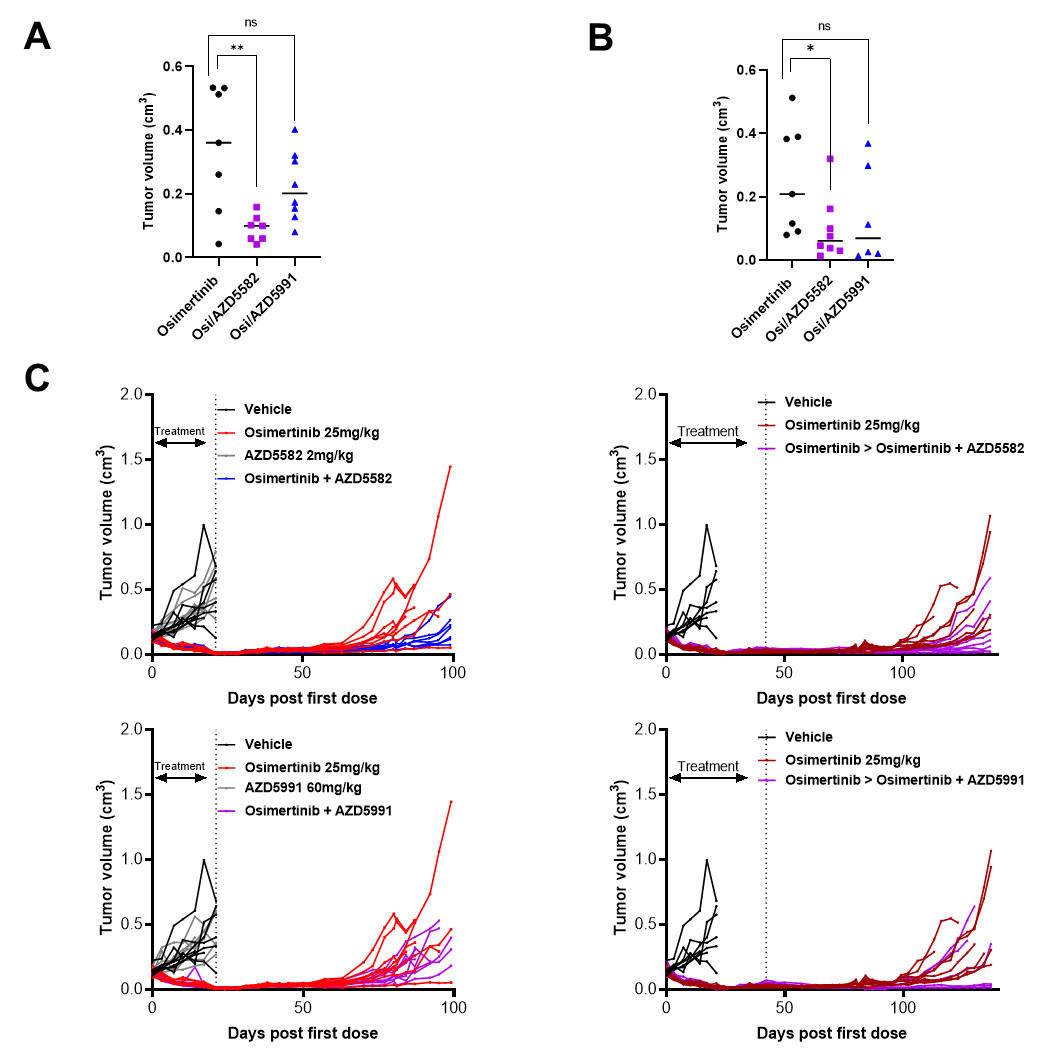
**

**Figure S4: SMAC mimetics inhibit DTP growth in vivo. A)** Plot of individual tumour volumes at day 87 post the initiation of dosing for the indicated up-front treatment groups. **p<0.01, NS = not significant; two-tailed t-test. **B)** Plot of individual tumour volumes at day 123 post the initiation of dosing for the indicated delayed combination treatment groups. *p<0.05, NS = not significant; two-tailed t-test. **C)** Growth curves of individual animals bearing PC9 xenograft tumours corresponding to the data in Figure 4. Two-tailed t-tests were used for statistical analyses. ***p<0.001, **p<0.01, *p<0.05, NS = not significant.

**
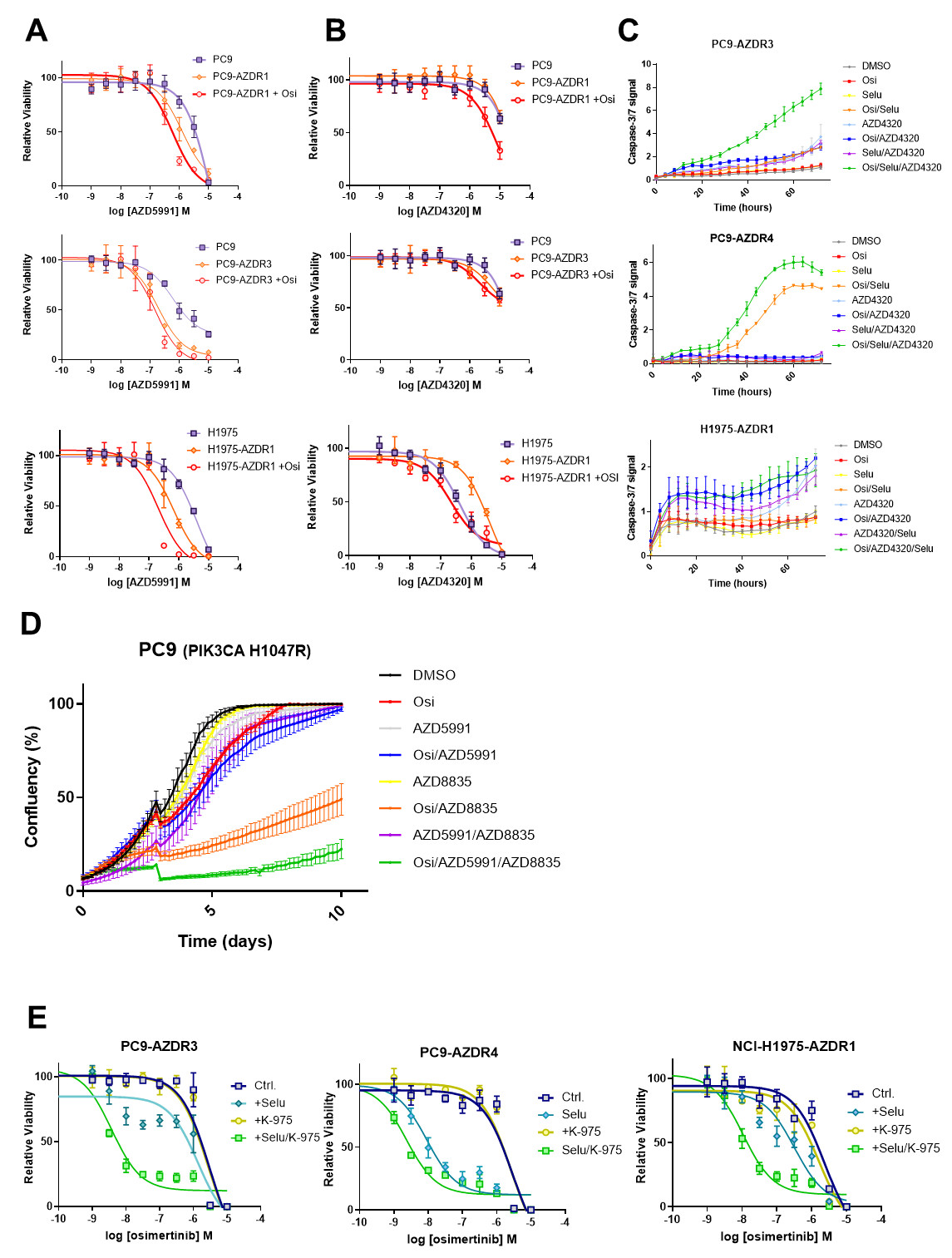
**

**
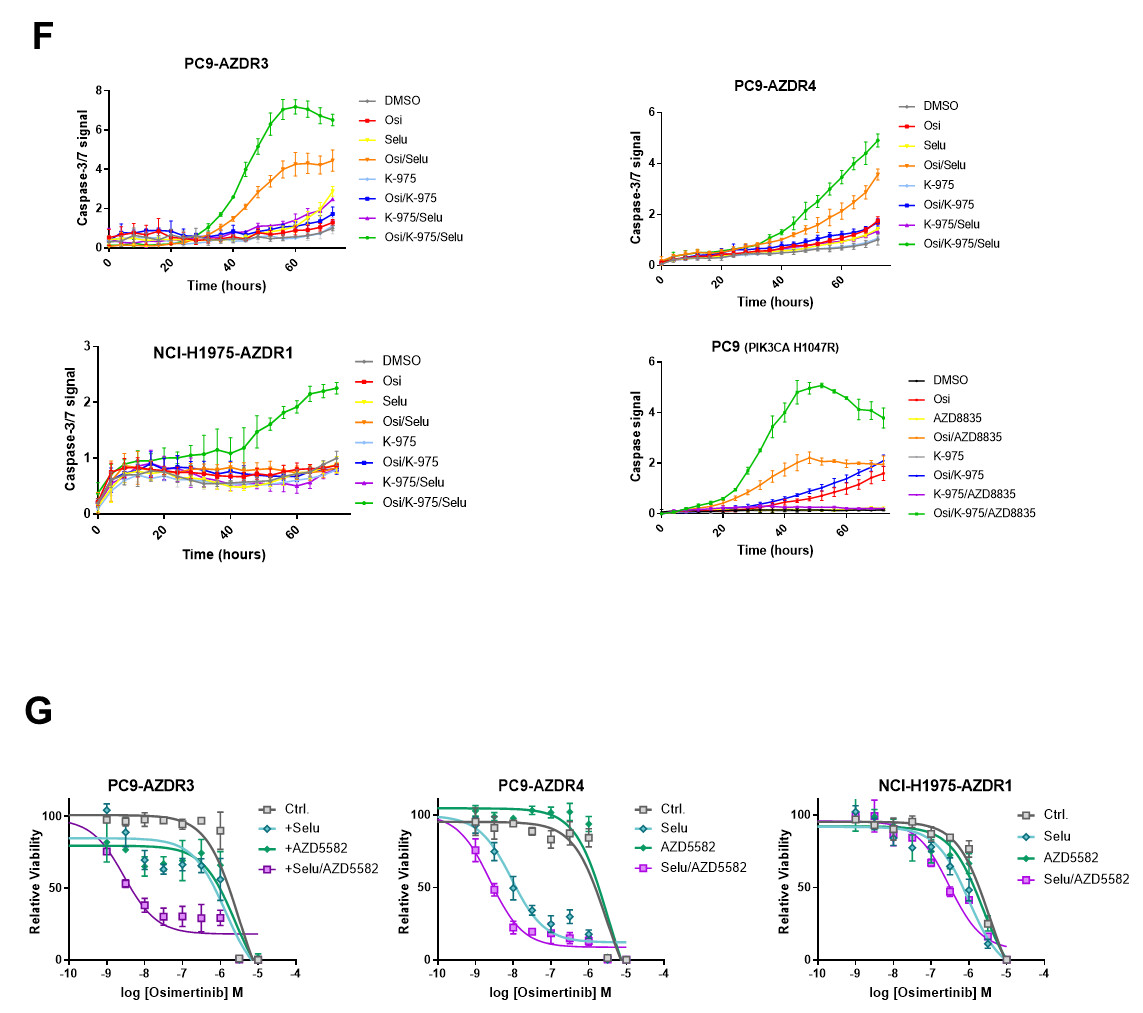
**

**Figure S5: BH3 and SMAC mimetics enhance apoptosis induced by agents targeting resistance drivers. A)** AZD5991 and **B)** AZD4320 dose response curves in the indicated cell lines as measured by Cell Titer Glo. Resistant cells were treated with or without a single dose of osimertinib (160 nM). **C)** Caspase-3/7 activity in the indicated cell lines for 72h with the indicated compounds (osimertinib 160 nM; selumetinib 300 nM; AZD4320 300 nM), compared to control. Osimertinib dose response curves in **D)** PC9 cells engineered to express the PIK3CA (H1047R) mutation, were treated with the indicated compounds and their growth monitored over 10 days using the Incucyte imaging platform to measure confluence. Osimertinib 500 nM; AZD5991 300 nM; AZD8835 1 μM. **E)** Osimertinib dose-response curves in PC9-AZDR3, PC9-AZDR4 and NCI-H1975-AZDR1 cells, co-treated with a single dose of the indicated compounds (selumetinib 300 nM; K-975 100 nM) alone or in combination, as measured by Cell Titer Glo. **F)** Caspase-3/7 activation assay in PC9-AZDR3, PC9-AZDR4, NCI-H1975-AZDR1 and PC9 (PIK3CA H1047R) cells, co-treated with a single dose of the indicated compounds (osimertinib 160 nM; selumetinib 300 nM; AZD8835 1 μM; K-975 100 nM), as indicated. **G)** Osimertinib dose-response curves in PC9-AZDR3, PC9-AZDR4 and NCI-H1975-AZDR1 cells, co-treated with a single dose of the indicated compounds (selumetinib 300 nM; AZD5582 100 nM) alone or in combination, as measured by Cell Titer Glo.

**
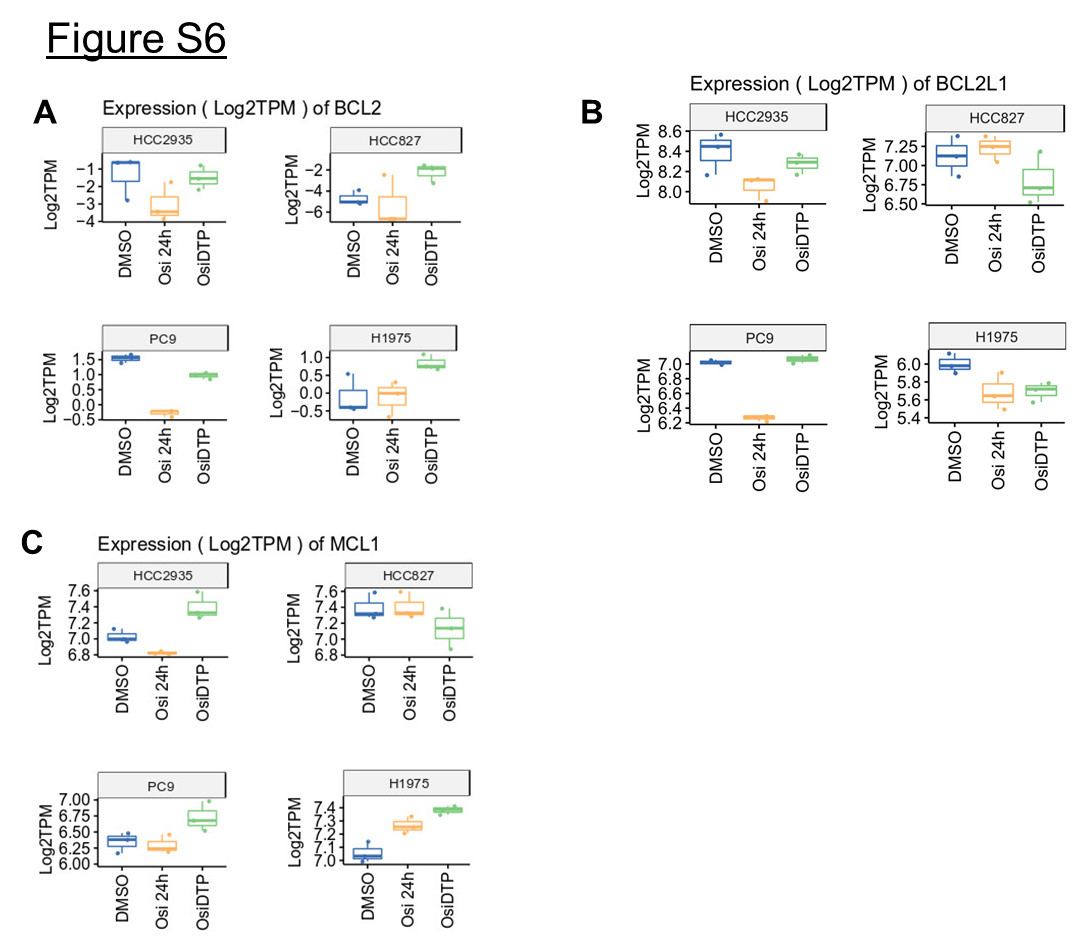
**

**Figure S6: Measuring transcript levels for anti-apoptotic genes in osimertinib DTPs.** mRNA expression of **A)** *BCL2* **B)** *BCL2L1* and **C)** *MCL1* transcripts taken from RNAseq dataset in panel of EGFRm cell lines treated acutely (24h) or chronically (DTP; 21d) with 500 nM osimertinib.
